# Transcriptomics Analysis Reveals a More Refined Regulation Mechanism of Methylation in a Drought-Tolerant Variety of Potato

**DOI:** 10.3390/genes13122260

**Published:** 2022-11-30

**Authors:** Zhenzhen Bi, Yihao Wang, Pengcheng Li, Chengju Li, Yindu Liu, Chao Sun, Panfeng Yao, Yuhui Liu, Zhen Liu, Jiangping Bai

**Affiliations:** 1 State Key Laboratory of Aridland Crop Science (Gansu Agricultural University), Lanzhou 730070, China; 2 College of Agronomy, Gansu Agricultural University, Lanzhou 730070, China

**Keywords:** potato, methylation, drought resistance, transcriptomics

## Abstract

Whether DNA methylation modification affects the gene transcription and expression of potatoes under drought stress is still unknown. In this study, we used comparative transcriptomics to explore the expression pattern of related genes of the drought-tolerant variety Qingshu 9 (Q) and the drought-sensitive variety Atlantic (A) under drought stress and DNA methylation inhibitor treatment. The results showed that there was a significant difference in the number of DEGs between the two varieties’ responses to mannitol and 5-azad C, especially when they were co-treated with two reagents, and the gene expression of Q was more sensitive to mannitol after two hours. Furthermore, we found that these differentially expressed genes (DEGs) were significantly enriched in DNA replication, transcription, translation, carbohydrate metabolism, photosynthesis, signal transduction, and glutathione metabolism. These results indicate that the difference in the background of methylation leads to the difference in drought resistance of the two varieties. The complexity of the DNA methylation of variety Q might be higher than that of variety A, and the method of methylation regulation is more refined. This study systematically expands the understanding of the molecular mechanism wherein DNA methylation regulates the response to drought stress.

## 1. Introduction

The response of plants to drought stress is a complex biological process that is jointly regulated by a variety of signaling pathways [1]. When suffering from drought stress, plants will activate the downstream functional gene expression through signal transduction, including kinases, hormones, calcium ions, transcription factors, etc. [2,3]. In recent years, more and more studies have shown that epigenetic regulation plays an important role in the response of plants to drought stress [4,5]. DNA methylation is a kind of epigenetic modification, and plant DNA methylation functions in biological processes such as chromatin inhibition, cell differentiation, gene expression regulation, and plant growth and development [6]. In addition, abiotic stress can cause changes in the degree of DNA methylation during plant development, and in turn, DNA methylation is also involved in plants’ response to abiotic stress [7,8]. Therefore, it is particularly important to clarify the impact of drought stress on the changes in DNA methylation.

After drought stress, HUW468 showed hypermethylation in the whole genome, while C306 showed hypomethylation in the whole genome [9]. In *Populus tomentosa*, the proportion of cytosine methylation under drought stress is 10.04% and only 7.75% under watering treatment [10]. The same pattern was noted in drought-sensitive rice varieties, and the DNA methylation level increased under drought [11]. Under drought stress, methylation 100 bp upstream of the transcription start site (TSS) of *Populus tomentosa* inhibited gene expression [10]. The above results indicate that transcription factors may play an important role in the drought stress response of *P. tomentosa* through changes in DNA methylation.

The different tolerances to salt and drought among the three rice varieties are related to the existence of differentially methylated regions [12]. Barley has a high and stable level of whole-genome DNA methylation under water-shortage conditions, but there is a demethylation trend in the leaves and a methylation trend in the roots [13]. During the drought-recovery process, DNA methylation and demethylation occurred simultaneously in the leaves of *Boea hygrometirica* but were dominated by demethylation [14]. In perennial ryegrass, the methylation level was 57.67% under normal conditions and decreased to 47.39% under drought stress [15]. As for *Macrotyloma uniflorum*, the methylation degree of drought-sensitive HPKC2 (10.1%) was higher than that of drought-tolerant HPK4 (8.6%) [16]. A comparative analysis of the differential methylation sites showed that these genes are mainly involved in plant signal transduction, material and energy metabolism, plant growth and development, stress response, and other physiological reaction processes [17].

Sulfite sequencing analysis showed that compared with the control, the DNA methylation level of the *AtGSTF14* promoter region was significantly reduced, and its expression levels increased significantly in the drought-treated Arabidopsis plants [18]. Under drought stress treatment, the level of genomic DNA methylation of pea was significantly increased, thereby inhibiting gene expression from adapting to drought [19]. There is a difference in genome-wide DNA methylation between drought-tolerant (Bachar) and drought-sensitive (F177) *Vicia faba* genotypes. However, drought stress leads to a decrease in Bachar and F177 genomic DNA methylation levels, while the Bachar demethylation level is higher than that of F177 [20]. Furthermore, six differentially methylated regions related to drought stress were identified, including genes encoding lipoxygenase (LOX), calcium-dependent protein kinase (CDPK), the ABC transporter family (ABC), glycosyl hydrolase (GH), and phosphoenolpyruvate carboxylase (PEPC). Under drought stress, the expression levels of *VfLOX*, *VfCDPK*, *VfABC,* and *VfGH* genes in Bachar are higher than that of F177. More importantly, the DNA methylation changes caused by drought resistance can be inherited by the offspring, that is, stress memory, which has been confirmed in *Arabidopsis* [21], rape [22], and rice [23]. These indicate that the DNA methylation of plants is heritable and may contribute to improving their drought resistance, which may have significance in plant breeding.

5-Aza-2′-deoxycytidine (5-azad C) is commonly used in plants to inhibit the degree of DNA methylation [24]. 5-azad C is an analog of cytosine and binds irreversibly to DNA methyltransferase, which makes it difficult for the genome to maintain the methylation status [25,26]. It was found that the DNA demethylation treatment seriously affected the growth and development of the potato variety of Atlantic test-tube seedlings [27]. In wheat, 5-azacytidine can reduce the level of wheat genomic DNA methylation, thereby improving salt tolerance [28]. Treatment of cabbage seedlings and rice seeds showed abnormal phenomena, such as dwarf plants and small leaves after growth and maturity, and the degree of genomic DNA methylation was measured and showed a significant reduction [29]. Therefore, the function of DNA methylation is complex in plants.

The lack of water in potatoes (*Solanum tuberosum*) seriously affected the normal growth and development of potato plants [30]. At present, research on potato DNA methylation mainly focuses on the changes in DNA methylation under in vitro culture and stress environment [31,32,33]. In our previous work, we found that DNA demethylation under drought stress is involved in regulating the phenotypic traits and physiological and biochemical responses of potatoes [34]. In this study, comparative transcriptomics analysis was used to explore the expression characteristics of related genes in the drought-tolerant variety Qingshu 9 (Q) and drought-sensitive variety Atlantic (A) under drought stress and DNA methylation inhibitors. We hope to clarify the molecular mechanism by which DNA methylation participates in the regulation of potato response to drought stress, and promote potato drought resistance molecular breeding.

## 2. Materials and Methods

### 2.1. Plant Cultivation and Treatment 

The drought-tolerant potato variety used was Q, and the drought-sensitive variety was A. We can use the paper bridge method to inoculate the stems of two varieties of potato tissue cultured seedlings in MS liquid medium (without agar); each stem has an axillary bud, and the test-tube seedling culture environment condition is 22 (±2) °C. The light intensity is 25–37.5 μmol m^−^^2^ s^−^^1^ and the light duration is 16 h every day. After 24 days of cultivation, the potato test-tube plantlets were treated with three treatment methods; namely, 200 mmolL-1 mannitol treatment (simulated drought treatment and referred to as M in the following), 60 μmol L-1 5-azadC treatment (demethylation and referred to as A in the following), and the two reagents were treated simultaneously (referred to as D in the following). After the treatment, the stems and leaves of the test tube seedlings were treated for 2 h, 6 h, 12 h, and untreated (control, 0 h), quickly frozen with liquid nitrogen, and stored in an ultra-low temperature refrigerator at −80 °C for later use. Three biological replicates were used for each treatment. 5-azadC (5-aza-2′-deoxycytidine) was purchased from Sigma-Aldrich.

### 2.2. RNA Extraction, Library Construction, and Transcriptome Sequencing

First, the total RNA of 60 samples (2 varieties × (control + (3 treatments × 3 time points)) × 3 biological replicates) were extracted using Trizol reagent (Invitgen, Waltham, MA, USA). Then, total RNA was treated using Turbo DNase I (Ambion, Austin, TX, USA) for 30 min and purified using RNeasy^®^ Plant Mini Kit (QIAGEN, Hilden, Germany). Then, the TruSeq RNA sample Prep V2 kit (Illumina, San Diego, CA, USA) was used to construct a transcriptome-sequencing cDNA library and the Agilent 2100 TapeStation (Agilent, Santa Clara, CA, USA) system was used to detect the quality of the cDNA library. Finally, paired-end sequencing was performed on the Illumina NovaSeq 6000 sequencer by Beijing Biomics Biotech Co., Ltd. (Beijing, China). After the sequencing was completed, we uploaded the data to the NCBI database under the BioProject number PRJNA783478. 

### 2.3. Transcriptome Data Analysis

First, we used FASTQC (https://www.bioinformatics.babraham.ac.uk/projects/fastqc/, accessed on 6 September 2021) to perform quality control on the off-machine data and calculate the Q20, Q30, and GC content. The potato reference genome sequence was downloaded from the ENSEMBL plant database (http://solanaceae.plantbiology.msu.edu/, accessed on 6 September 2021). We used TopHat2 [35] to compare Clean Reads with the reference genome to obtain the position information on the reference genome or gene, as well as the unique sequence feature information of the sequenced sample. We used the Cuffquant and Cuffnorm components of the Cufflinks (https://github.com/cole-trapnell-lab/cufflinks, accessed on 6 September 2021) software to quantify the transcript and gene-expression level through the location information of Mapped Reads on the gene. 

Then, we used HISAT2 (http://daehwankimlab.github.io/hisat2/, accessed on 6 September 2021) to compare sequencing reads to the potato reference genome to obtain sam files. We used Samtools (https://github.com/samtools/samtools, accessed on 6 September 2021) to convert sam files into bam files and sort them, used Cufflinks to assemble and quantify the sorted bam files, used HTseq-count to calculate the counts of each sample transcript, used Python script to calculate the length of each gene in the reference genome, and used the R package Dseq2 to calculate the FPKM (fragments per kilobase of transcript per million fragments mapped) of each gene and analyze the differential expression between samples. Corrected *p* < 0.01 and a multiple of change greater than 2 were defined as indicating differentially expressed genes (DEGs). Pearson’s correlation coefficient was used as the evaluation index of biological repetition correlation, and the correlation between samples was statistically mapped [36]. The closer r was to 1, the stronger the correlation between the two repetitive samples. 

Next, we used the online tool:Profiler (https://biit.cs.ut.ee/gprofiler_beta/gost, accessed on 12 October 2021) to perform COG, GO, and KEGG functional annotation and enrichment analysis of differentially expressed genes, and *p* < 0.05 as the significant enrichment GO term. Additionally, we used KEGG pathway screening conditions. We used the interaction relationship in the STRING protein interaction database (http://string-db.org/, accessed on 12 October 2021) to analyze the differential gene protein interaction network. 

Based on the TopHat2 (http://ccb.jhu.edu/software/tophat/index.shtml, accessed on 12 October 2021) alignment results of the reads of each sample and the reference genome sequence, GATK [37] software was used to identify single-base mismatches between the sequenced samples and the reference genome and potential SNP sites and InDel (insertion–deletion). 

### 2.4. Promoter Methylation CpG Island Prediction and Cis-Elements Analysis of Important DEGs

We first use the perl script to extract the 1500 bp promoter sequence upstream of the analyzed gene in batches and then the online software MethPrimer (http://www.urogene.org/cgi-bin/methprimer/MethPrimer.cgi, accessed on 12 October 2021) to regulate the differentially enriched gene. 5′ CpG island prediction was performed in the area, and the prediction parameters were set as: CpG island length > 100 bp, CPG detection content/CpG expected content (Obs/Exp) > 0.60, and GC content > 50%. We used Plant Care (http://bioinformatics.psb.ug ent.be/webtools/plantcare/html/, accessed on 12 October 2021) to predict the cis-acting elements of gene promoters.

### 2.5. Real-Time qPCR Verification

Primer Express 3.0.1 was used to design primers for the 20 selected DEGs (Supplementary Table S1), and then we performed real-time fluorescent quantitative PCR analysis. The RNA of three biological replicates of the selected sample (after 6 h of three kinds of treatment) was reverse-transcribed into cDNA using a cDNA synthesis kit (TaKaRa, Kyoto, Japan), actin was used as an internal reference, and qPCR was performed using the QuantStudio5 real-time fluorescent quantitative PCR system (ABI, California, USA)—PCR detection. Three biological replicates of each gene were used in each sample, and the relative expression level was calculated according to the 2^−ΔΔCT^ method. 

## 3. Results

### 3.1. Transcriptome Sequencing and Functional Annotation and Identification of the DEGs

As shown in the Supplementary Materials, Table S2, after data filtering and quality evaluation, 60 samples produced approximately 410 G of clean data, and each sample produced approximately 6.8 G of effective data on average. The GC content of each sample was in the range of 42.15~47.82%, the Q20 value was above 97%, and the Q30 value was above 91.98%. At the same time, the unique comparison rate between the reads of each sample and the reference genome sequence was over 98%. A total of 33,285 genes were detected in all samples, of which 29,689 genes (Supplementary Materials Table S3) had effective expression values (FPKM), and functional annotations were made for these genes (Supplementary Materials, Tables S4 and S5). In this study, 9876 DEGs were identified from the pairwise comparison combinations of 20 treatments (60 samples) (Supplementary Materials, Tables S6–S9). The PCA analysis (Supplementary Materials, Figure S1) and heat map for all samples indicate that biological repeats reached their expected effect (Supplementary Materials, Figures S2–S4). 

#### 3.1.1. The Number of DEGs in the Drought-Sensitive Variety A

**Figure 1:**
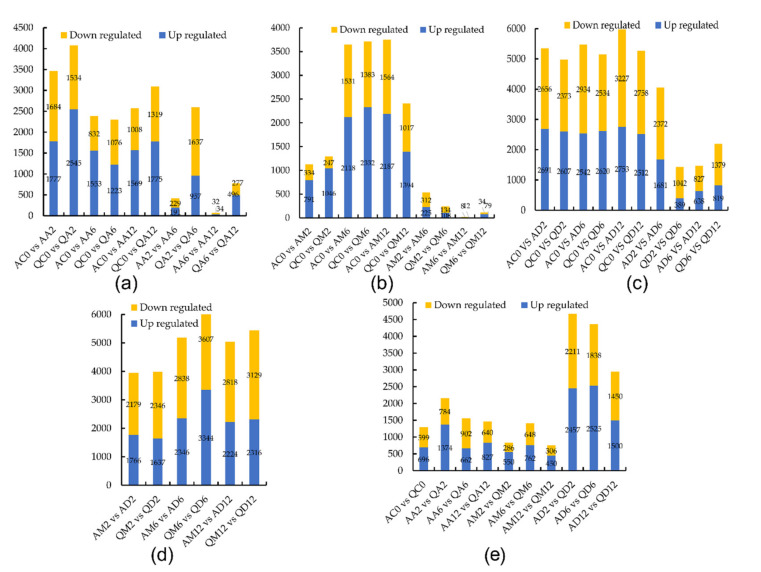
Differentially expressed genes (DEGs) statistics for comparisons. (**a**) Number of DEGs in varieties Atlantic (A) and Qingshu 9 (Q) under methylation inhibitor treatment. (**b**) Number of DEGs in varieties A and Q under simulated drought treatment. (**c**) Number of DEGs in varieties A and Q under dual treatment. (**d**) Number of DEGs in comparison between simulated drought and dual treatment. (**e**) Number of DEGs among varieties A and Q under three treatments.

Under the conditions of methylation inhibitor treatment, after 2 h of treatment (Figure 1a), there were 3461 DEGs, of which 1777 were upregulated and 1684 were downregulated. After 6 h, there were 2385 DEGs, of which 1553 were upregulated and 832 were downregulated. After 12 h of treatment, there were 2577 DEGs, of which 1569 were upregulated and 1008 were downregulated genes. Compared with the results after 2 h, there were 420 DEGs, of which 191 were upregulated and 229 were downregulated after 6 h of treatment. Compared with results after 6 h, there were 66 DEGs, of which there were 34 upregulated and 32 downregulated expressed genes after 12 h of treatment.

Under simulated drought conditions, after 2 h of treatment (Figure 1b), there were 1125 DEGs, of which 791 were upregulated and 334 were downregulated. After 6 h of treatment, there were 3649 DEGs, of which 2118 were upregulated and 1531 were downregulated. After 12 h of treatment, there were 3751 DEGs, including 2187 upregulated genes and 1564 downregulated genes. Compared with treatments for 6 h and 2 h, there were 537 DEGs, of which 225 were upregulated and 312 were downregulated. Compared with treatment for 6 h, there were 20 DEGs, of which 12 were upregulated and 8 were downregulated after 12 h of treatment.

Under the dual treatment conditions, after 2 h of treatment (Figure 1c), there were 5347 DEGs, of which 2691 were upregulated and 2656 were downregulated. After 6 h of treatment, there were 5476 DEGs, of which 2542 were upregulated and 2934 were downregulated. After 12 h of treatment, there were 5980 DEGs, including 2753 upregulated genes and 3227 downregulated genes. Compared with treatment for 6 h and 2 h, there were 4053 DEGs, of which 1681 were upregulated genes and 2371 were downregulated genes. Compared with results after 6 h, there were 1465 DEGs, of which 638 were upregulated and 827 were downregulated after 12 h of treatment.

Compared with the simulated drought treatment, under the dual treatment conditions (Figure 1d), after 2 h of treatment, there were 3955 DEGs, of which 1766 were upregulated and 2179 were downregulated. After 6 h of treatment, there were 5184 DEGs, of which 2346 were upregulated and 2838 were downregulated. After 12 h of treatment, there were 5042 DEGs, of which 2224 were upregulated and 2818 were downregulated.

**Figure 2:**
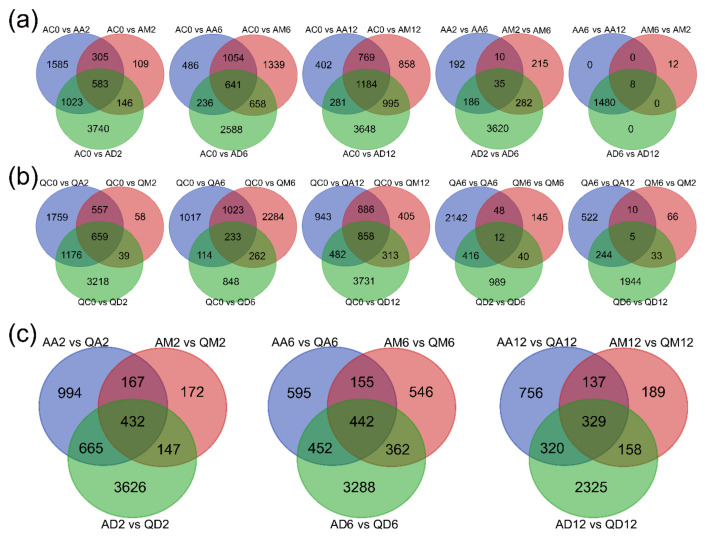
Venn diagram of DEGs distributed in every comparison. (**a**) Statistics of DEGs distributed among methylation inhibitor, simulated drought, and dual treatment in variety A. (**b**) Statistics of DEGs distributed among methylation inhibitor, simulated drought, and dual treatment in variety Q. (**c**) Statistics of DEGs distributed among three kinds of treatments in variety A and Q.

A Venn diagram was used to count the common DEGs and specific DEGs under different treatment conditions. After 2 h of treatment (Figure 2a), there were 583 DEGs shared by the three kinds of treatment, 1585 unique DEGs for methylation inhibitors, 109 unique DEGs for simulated drought, and 3740 unique DEGs for dual treatments. After 6 h of treatment, there were 641 DEGs shared by three kinds of treatment, 486 unique DEGs for methylation inhibitors, 1339 unique DEGs for drought treatment, and 2588 DEGs for double dry treatment. After 12 h of treatment, there were 1184 DEGs shared by three kinds of treatment, 402 unique DEGs for methylation inhibitors, 858 unique DEGs for simulated drought treatment, and 3648 DEGs for dual treatments. Compared with treatment for 6 h and 2 h, there were 35 DEGs shared by three kinds of treatment, 192 specific DEGs for methylation inhibitors, 215 specific DEGs for simulated drought treatment, and 3620 DEGs for dual treatments. Compared with treatments for 12 h and 6 h, there were only eight DEGs shared by three kinds of treatment; there were no specific DEGs for the methylation inhibitor and dual treatment, and the number of specific DEGs of the simulated drought treatment was 12.

#### 3.1.2. The Number of DEGs in Drought-Tolerant Potato Variety Q

Under the conditions of methylation inhibitor treatment (Figure 1a), after 2 h, there were 4079 DEGs, of which 2545 were upregulated and 1534 were downregulated. After 6 h, there were 2899 DEGs, of which 1223 were upregulated and 1676 were downregulated. After 12 h of treatment, there were 3094 DEGs, of which 1775 were upregulated and 1319 were downregulated. Compared with treatments for 6 h and 2 h, there were 2594 DEGs, of which 957 were upregulated and 1637 were downregulated. Compared with treatments for 12 h and 6 h, there were 773 DEGs, of which 496 were upregulated and 277 were downregulated.

Under simulated drought conditions (Figure 1b), after 2 h of treatment, there were 1293 DEGs, of which 1046 were upregulated and 247 were downregulated. After 6 h of treatment, there were 3715 DEGs, of which 2332 were upregulated and 1383 were downregulated. After 12 h, there were 2411 DEGs, of which 1394 were upregulated and 1017 were downregulated. Compared with the treatments for 6 h and 2 h, there were 242 DEGs, of which 108 were upregulated and 134 were downregulated. Compared with the treatments for 12 h and 6 h, there were 113 DEGs, of which 79 were upregulated and 34 were downregulated.

Under the dual-treatment stacking condition (Figure 1c), after 2 h of treatment, there were 4980 DEGs, of which 2607 were upregulated and 2373 were downregulated. After 6 h, there were 5154 DEGs, of which 2620 were upregulated and 2534 were downregulated. After 12 h, there were 5270 DEGs, of which 2512 were upregulated and 2758 were downregulated. Compared with the treatments for 6 h and 2 h, there were 1431 DEGs, of which 389 were upregulated and 1042 were downregulated. Compared with the treatments for 12 h and 6 h, there were 2198 DEGs, of which 819 were upregulated and 1379 were downregulated. 

Compared with the simulated drought treatment (Figure 1d), under the dual treatment condition, after 2 h, there were 3983 DEGs, of which 1637 were upregulated and 2346 were downregulated. After 6 h, there were 6951 DEGs, of which 3344 were upregulated and 3607 were downregulated. After 12 h, there were 5445 DEGs, of which 2316 were upregulated and 3129 were downregulated. 

A Venn diagram counted the common and specific DEGs under different treatment conditions (Figure 2b). After 2 h, there were 659 DEGs shared by three kinds of treatment, 1759 DEGs were specific for treatment with methylation inhibitors, 3218 DEGs were specific for dual treatments, and 58 DEGs were specific for simulated drought treatment. After 6 h, there were 233 DEGs shared by three kinds of treatment, 1017 unique DEGs for treatment with methylation inhibitors, and 848 unique DEGs for treatment with dual treatments. There were 2284 DEGs unique to simulated drought treatment. After 12 h, there were 858 DEGs shared by three kinds of treatment, 943 DEGs specific for treatment with methylation inhibitors, and 3731 DEGs specific for dual treatments. 

There were 405 DEGs unique to simulated drought treatment. Compared with treatment for 6 h and 2 h, there were 12 DEGs shared by three kinds of treatment, 2142 specific DEGs treated with methylation inhibitors, and 989 DEGs treated with dual treatments. There were 145 DEGs unique to simulated drought treatment. Compared with treatment for 12 h and 6 h, there were 5 DEGs shared by three kinds of treatment, 522 specific DEGs treated with methylation inhibitors, and 1944 DEGs treated with dual treatments. There were 66 DEGs unique to simulated drought treatment.

#### 3.1.3. The Number of DEGs of the Two Varieties

Under the untreated control condition (Figure 1e), there were 1295 DEGs in common in the two varieties, of which 696 were upregulated and 599 were downregulated. After 2 h of methylation inhibitor treatment, there were 2518 DEGs in common in the two varieties, of which 1734 were upregulated and 784 were downregulated. After 6 h, there were 1564 DEGs in common in the two varieties, of which 662 were upregulated and 902 were downregulated. After 12 h, there were 1467 DEGs in common in the two varieties, of which 827 were upregulated and 640 were downregulated. 

After 2 h of simulated drought treatment (Figure 1e), there were 636 DEGs in common in the two varieties, of which 350 were upregulated and 286 were downregulated. After 6 h of treatment, there were 1410 DEGs in common in the two varieties, of which 762 were upregulated and 648 were downregulated. After 12 h of treatment, there were 756 DEGs in common in the two varieties, of which 450 were upregulated and 306 were downregulated. 

Under the double-treatment condition (Figure 1e), after 2 h, there were 4668 DEGs in common in the two varieties, of which 2457 were upregulated and 2211 were downregulated. After 6 h of treatment, there were 4363 DEGs in common in the two varieties, of which 2525 were upregulated and 1838 were downregulated. After 12 h of treatment, there were 2950 DEGs in common in the two varieties, of which 1500 were upregulated and 1450 were downregulated.

A Venn diagram was used to count the differentially expressed genes and specific differentially expressed genes under different treatment conditions. After 2 h of treatment (Figure 2c), there were 432 DEGs shared by three kinds of treatment, 994 specific DEGs treated with methylation inhibitors, and 3626 DEGs treated with dual treatments. There were 172 DEGs unique to simulated drought treatment. After 6 h of treatment, there were 442 DEGs shared by three kinds of treatment, 595 unique DEGs treated with methylation inhibitors, and 3288 DEGs treated with dual treatments. There were 546 DEGs unique to simulated drought treatment. After 12 h of treatment, there were 329 DEGs shared by three kinds of treatment, 756 unique DEGs treated with methylation inhibitors, and 2325 DEGs treated with dual treatments. There were 189 DEGs unique to simulated drought treatment.

#### 3.1.4. Venn Diagram Statistics of Each Treatment’s DEGs in the Two Varieties

**Figure 3:**
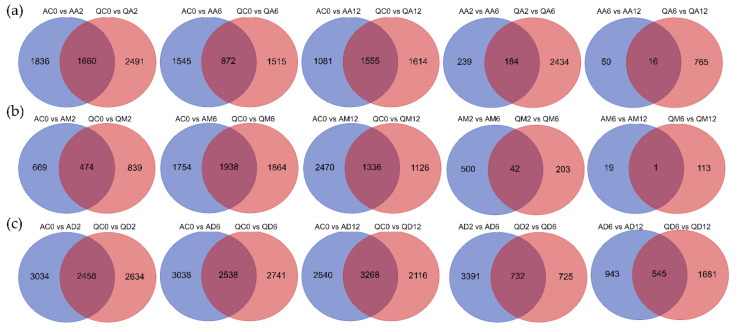
Venn diagram of DEGs distributed in comparisons. (**a**) Statistics of DEGs distributed in methylation inhibitor treatment between varieties A and Q. (**b**) Statistics of DEGs distributed in simulated drought treatment between varieties A and Q. (**c**) Statistics of DEGs distributed in dual treatment between varieties A and Q.

Under the methylation inhibitor treatment condition (Figure 3a) for 2 h, there were 1660 DEGs in common between the two varieties, 1836 DEGs specific to species A, and 2491 DEGs specific to species Q. After 6 h, there were 872 DEGs in common between the two varieties, 1545 DEGs specific to variety A, and 1515 DEGs specific to variety Q. After 12 h of treatment, there were 1555 DEGs shared between the two varieties, 1081 unique DEGs of variety A, and 1641 unique DEGs of variety Q. Compared with the treatment for 6 h and 2 h, there were 184 DEGs in common between the two varieties, 239 DEGs specific to variety A, and 2434 DEGs specific to variety Q. Compared with the treatment for 12 h and 6 h, there were 16 DEGs in common between the two varieties, 50 DEGs unique to variety A, and 765 unique DEGs to variety Q.

Under the simulated drought treatment (Figure 3b) for 2 h, there were 474 DEGs in common between the two varieties, 669 unique DEGs of variety A, and 839 unique DEGs of variety Q. After 6 h of treatment, there were 1938 DEGs shared between the two varieties, 1754 unique DEGs of variety A, and 1864 unique DEGs of variety Q. After 12 h of treatment, there were 1336 DEGs in common between the two varieties, 2470 unique DEGs of variety A, and 1126 unique DEGs of variety Q. Compared with 6 h of treatment and 2 h of treatment, there were 42 DEGs shared between the two varieties, 500 specific DEGs specific to variety A, and 203 specific DEGs specific to variety Q. Compared with the treatment for 12 h and 6 h, there was only 1 DEG shared between the two varieties, 19 DEGs specific to variety A, and 113 DEGs specific to variety Q. 

Under the condition of double treatment (Figure 3c), after 2 h, there were 2458 DEGs in common between the two varieties, 3034 DEGs unique to variety A, and 2634 DEGs unique to variety Q. After 6 h of treatment, there were 2538 DEGs in common between the two varieties, 3038 DEGs unique to variety A, and 2741 DEGs unique to variety Q. After 12 h of treatment, there were 3268 DEGs in common between the two varieties, 2840 unique DEGs of variety A, and 2116 unique DEGs of variety Q. Compared with the treatments for 6 h and 2 h, there were 732 DEGs in common between the two varieties, 3391 DEGs unique to variety A, and 725 unique DEGs to variety Q. Compared with the treatments for 12 h and 6 h, there were 545 DEGs in common between the two varieties, 943 DEGs specific to variety A, and 1681 DEGs specific to variety Q.

### 3.2. Functional Annotation Enrichment of DEGs

**Figure 4:**
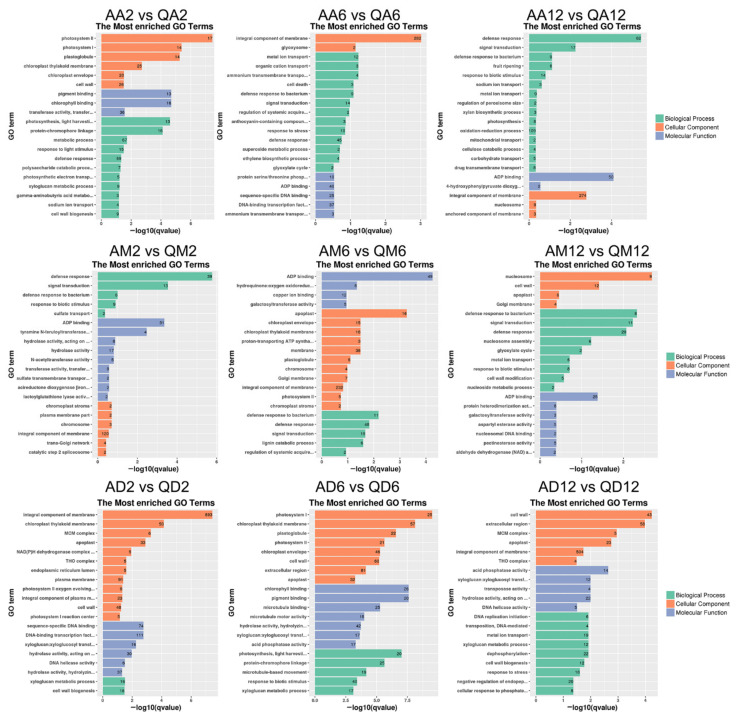
Annotation and functional classification of DEGs’ GO term. Histogram of most enriched GO classifications of potato DEGs under treatment. The ordinate is the enriched GO term; the abscissa is the enriched q value of the term as well as the number of differential genes on the column. Different colors distinguish biological processes, cellular components, and molecular functions.

**Figure 5:**
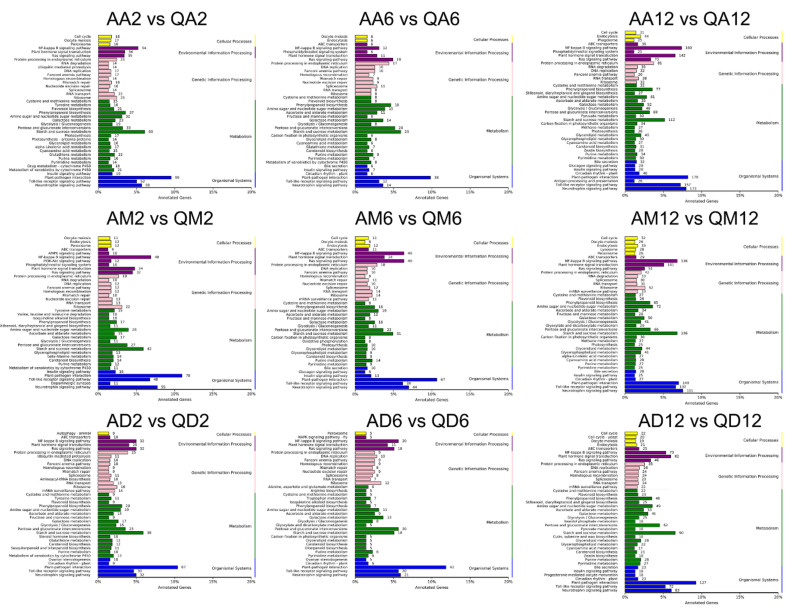
Annotation and functional enrichment of DEGs in KEGG database. Histogram of the most enriched KEGG pathway of potato DEGs under treatment. The ordinate is the enriched KEGG pathway; the abscissa is the number of DEGs on the column. Different colors distinguish cellular processes, environmental information processing, genetic information processing, metabolism, cellular components, and organismal systems.

**Figure 6:**
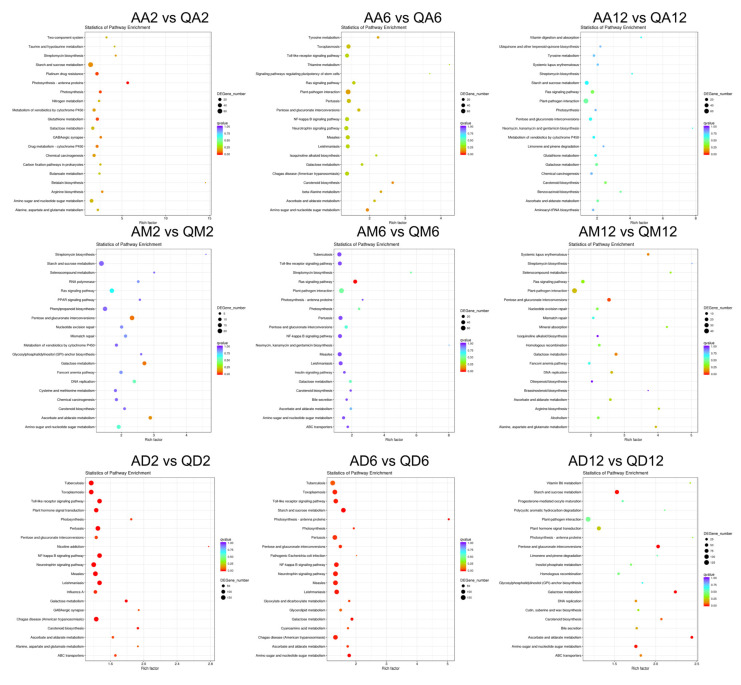
Functional enrichment of DEGs in KEGG database. Histogram of the most significantly enriched KEGG pathway of potato DEGs under treatment. The ordinate is the enriched KEGG pathway; the abscissa is the number of DEGs on the column. Different colors distinguish different significances. Red represents extremely prominent and blue represents generally significant. The diameter of the circle represents the number of enriched DEGs.

To fully analyze the functions of DEGs, we performed GO and KEGG functional enrichment analysis (Figure 4, Figure 5 and Figure 6; Supplementary Materials, Figures S5–S11). Under untreated conditions, in the GO term (Supplementary Materials, Figure S5b), DEGs between drought-sensitive varieties and drought-tolerant varieties were mainly enriched in the defense response to bacteria (12), defense response (48), ADP binding (41), signal transduction (13), and protein folding (11). The annotated KEGG pathways mainly include plant hormone signal transduction (28), protein processing in the endoplasmic reticulum (40), plant–pathogen interaction (52), pentose and glucuronate interconversions (25), and starch and sucrose metabolism (26) (Supplementary Materials, Figure S5c,d). 

After 2 h of methylation inhibitor treatment (Figure 4), in the GO term, DEGs common between the two varieties were mainly enriched in photosystem II (17), photosystem I (14), cell wall (26), and defense response (69). After 6 h of treatment, DEGs were mainly enriched in the integral component of the membrane (282, 54% of the DEGs), defense response (45), response to stress (10), ADP binding (40), DNA-binding transcription factor activity (37), and sequence-specific DNA binding (25). After 12 h of treatment, DEGs were significantly enriched in the defense response (62) and ADP binding (50), followed by the integral component of the membrane (274) and signal transduction (17). 

After 2 h of drought treatment, DEGs were significantly enriched in the defense response (39), followed by signal transduction (13), ADP binding (31), and the integral component of the membrane (120). After 6 h of treatment, DEGs were significantly enriched in ADP binding (49), apoplast (16), defense response to bacteria (8), defense response (48), etc. After 12 h of treatment, DEGs were mainly enriched in the nucleosome (9), followed by the defense response to bacteria (8), signal transduction (11) and defense response (29), cell wall (12) and ADP binding (25), etc. 

After 2 h of dual treatment, DEGs concentrated in the integral component of the membrane (893, accounting for 52% of the DEGs), followed by the chloroplast thylakoid membrane (50), MCM complex (6), apoplast (33), sequence-specific DNA binding (74), DNA-binding transcription factor activity (111), xyloglucosyl transferase activity (11), etc. After 6 h of treatment, DEGs were significantly enriched in photosystem I and light harvesting in photosystem I (25 + 20), followed by the chloroplast thylakoid membrane (57), chlorophyll binding (26), photosystem II (21), protein–chromophore linkage (25), microtubule binding (25), chloroplast envelope (46), cell wall (60), etc. After 12 h of treatment, DEGs were mainly enriched in the cell wall (43), extracellular region (58), MCM complex (5), MCM complex (23), integral component of the membrane (540), acid phosphatase activity (14), processes related to DNA replication, including initiation (6) and DNA helicase activity (5), etc.

According to the enrichment results of the KEGG signaling pathway (Figure 5 and Figure 6, Supplementary Materials, Figures S6 and S7), after 2 h of methylation inhibitor treatment, the DEGs common between the two varieties were mainly enriched in photosynthesis (17 + 14), starch and sucrose metabolism (63), amino sugar and nucleotide sugar metabolism (32), and other pathways. After 6 h of treatment, the KEGG pathways annotated by DEGs mainly included: amino sugar and nucleotide sugar metabolism (28); carotenoid biosynthesis (14); plant–pathogen interaction (78); beta-alanine metabolism (14); tyrosine metabolism (15); ascorbate and aldarate metabolism (15); and pentose and glucuronate interconversions (27). After 12 h of treatment, the identified DEGs-enriched KEGG pathways mainly included: the Ras signaling pathway (32); carotenoid biosynthesis (12); galactose metabolism (17); benzoxazinoid biosynthesis (6); plant–pathogen interaction (67); and starch and sucrose metabolism (38).

After 2 h of drought treatment, the identified DEGs-enriched KEGG pathways mainly included: pentose and glucuronate interconversions (20); galactose metabolism (14); ascorbate and aldarate metabolism (11); DNA replication (10); amino sugar and nucleotide sugar metabolism (15); the Ras signaling pathway (19); and mismatch repair (9). After 6 h of treatment, the KEGG pathways to which DEGs belonged mainly included: the Ras signaling pathway (40); plant–pathogen interaction (67); photosynthesis (10); galactose metabolism (16); pentose and glucuronate interconversions (23); ascorbate and aldarate metabolism (12); and carotenoid biosynthesis (9). After 12 h of processing, the KEGG pathways annotated by DEGs mainly included: pentose and glucuronate interconversions (20); galactose metabolism (13); alanine, aspartate, and glutamate metabolism (6); plant–pathogen interaction (42); DNA replication (10); arginine biosynthesis (5); ascorbate and aldarate metabolism (9); selenocompound metabolism (4); mineral absorption (4); the Ras signaling pathway (18); homologous recombination (9); nucleotide excision repair (9); mismatch repair (8); and other metabolic pathways.

Under dual-treatment conditions, after 2 h of treatment, the KEGG pathways annotated by DEGs mainly included: plant hormone signal transduction (142); galactose metabolism (52); carotenoid biosynthesis (31); nicotine addiction (12); photosynthesis (26); pentose and glucuronate interconversions (69); ABC transporters (36); ascorbate and aldarate metabolism (35); alanine, aspartate, and glutamate metabolism (18); amino sugar and nucleotide sugar metabolism (61); carbon fixation in photosynthetic organisms (34); cyanoamino acid metabolism (27); circadian rhythm—plant (40); fatty acid elongation (112), and so on. After 6 h, the annotated KEGG pathways mainly included: photosynthesis—antenna proteins (24); starch and sucrose metabolism (136); amino sugar and nucleotide sugar metabolism (72); galactose metabolism (50); pentos and glucuronate interconversions (66); photosynthesis (25); ascorbate and aldarate metabolism (34); glyoxylate and dicarboxylate metabolism (29); cyanoamino acid metabolism (28); glycerolipid metabolism (44); diterpenoid biosynthesis (24); carotenoid biosynthesis (23); glycosylphosphatidylinositol (GPI)-anchor biosynthesis (14); ABC transporters (29); carbon fixation in photosynthetic organisms (30); apoptosis (17); mineral absorption (10); cutin, suberine, and wax biosynthesis (22); alpha-linolenic acid metabolism (27); glycerophospholipid metabolism (41); aminobenzoate degradation (12); alanine, aspartate, and glutamate metabolism (14); glycolysis/gluconeogenesis (41); methane metabolism (27); nitrogen metabolism (12); and the two-component system (7). After 12 h of treatment, the KEGG pathways annotated by DEGs mainly included: plant–pathogen interaction (127); starch and sucrose metabolism (90); plant hormone signal transduction (82); pentose and glucuronate interconversions (62); amino sugar and nucleotide sugar metabolism (49); the Ras signaling pathway (46); galactose metabolism (41); ascorbate and aldarate metabolism (33); glycerolipid metabolism (28); DNA replication (26); ABC transporters (25); stilbenoid, diarylheptanoid, and gingerol biosynthesis (25); homologous recombination (24); circadian rhythm—plant (23); carotenoid biosynthesis (21); cutin, suberine, and wax biosynthesis (18); inositol phosphate metabolism (18); pyruvate metabolism (18); cyanoamino acid metabolism (17); vitamin B6 metabolism (9); photosynthesis—antenna proteins (8); carbon fixation pathways in prokaryotes (8); and nitrogen metabolism (8).

### 3.3. Protein–Protein Interaction Network Analysis

**Figure 7:**
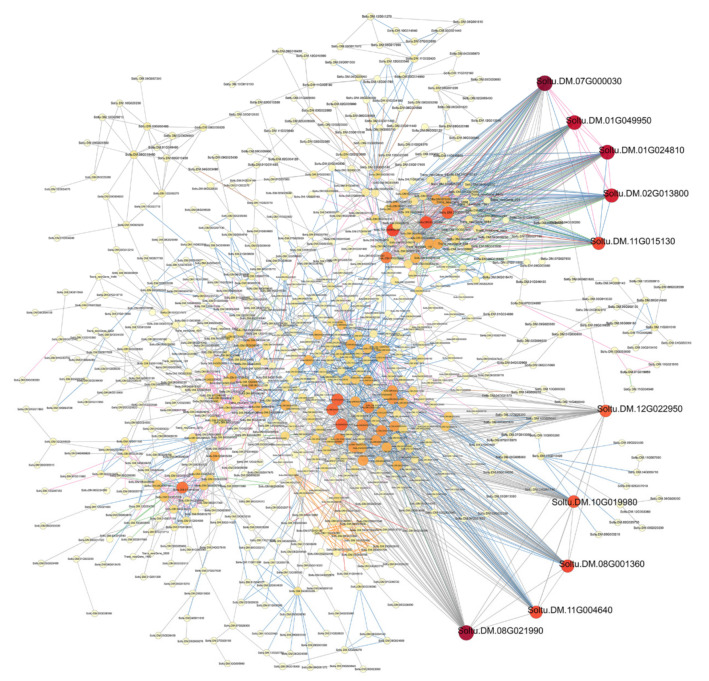
A PPI (protein–protein interaction) network of DEGs from variety A versus Q under dual treatment for 2 h. The interaction relationship in the STRING protein interaction database (http://string-db.org/ accessed on 6 September 2021) was then used to analyze the DEG coding protein interaction network. The ID of key hubs corresponding proteins were as follows: Soltu.DM.07G000030: un-known protein involved in defense mechanisms; Soltu.DM.08G021990: phosphoribulokinase; Soltu.DM.01G049950, Soltu.DM.11G015130, Soltu.DM.01G024810, and Soltu.DM.02G013800: DNA replication licensing factor MCM; Soltu.DM.08G001360: photosystem I reaction center subunit psaK; Soltu.DM.11G004640: malate dehydrogenase (NADP); and Soltu.DM.10G019980: fructose-1,6-bisphosphatase.

**Figure 8:**
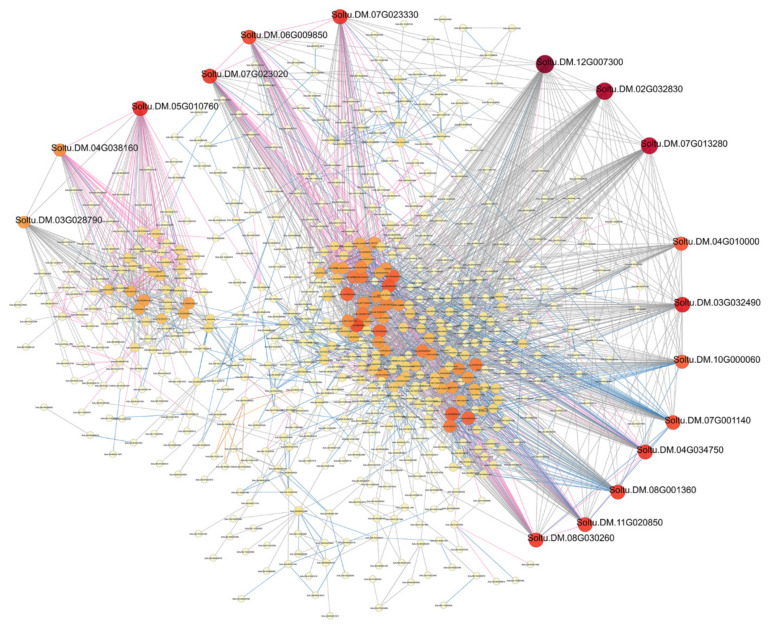
A PPI network of DEGs from variety A versus Q under dual treatment for 6 h. The interaction relationship in the STRING protein interaction database (http://string-db.org/ 6 September 2021) was then used to analyze DEG coding protein interaction network. The ID of key hubs corresponding proteins were as follows: Soltu.DM.12G007300: ATP synthase delta chain; Soltu.DM.02G032830: peptidylprolyl cist-rans isomerase CYP38; Soltu.DM.07G013280: peroxiredoxin Q; Sol-tu.DM.03G032490: magnesium protoporphyrin IX methyltransferase; Soltu.DM.07G023020: unknown protein; Soltu.DM.05G010760: mitotic spindle checkpoint protein MAD2; Soltu.DM.11G020850: 50S ribosomal protein L9; and Soltu.DM.08G030260 encoding 50S ri-bosomal protein L11; Soltu.DM.07G023330: 50S ribosomal protein L13; and Soltu.DM.08G001360: photosystem I reaction center subunit psaK.

**Figure 9:**
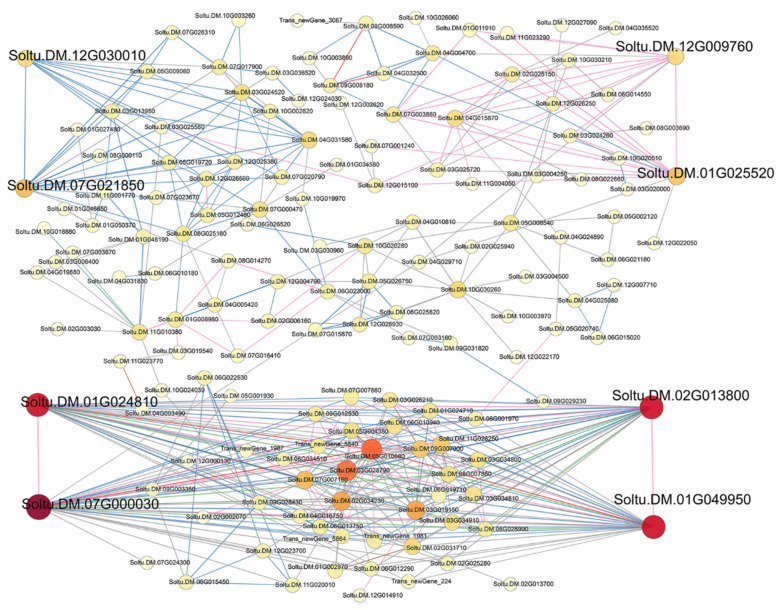
A PPI network of DEGs from variety A versus Q under dual treatment for 12 h.

We constructed an interaction network based on the DEGs retrieved from known PPI databases (Figure 7, Figure 8 and Figure 9, Supplementary Material, Figures S12–S17). In terms of comparisons between variety A and Q under control conditions, 689 DEGs can be linked via known relationships, including reaction, catalysis, binding, and inhibition (Supplementary Materials, Figure S12). Significantly, the Soltu.DM.08G017320 (17 links and was mainly upregulated), Soltu.DM.07G003860 (14 links and was mainly downregulated), and Soltu.DM.10G024790 (13 links and was mainly upregulated) encoding aldehyde dehydrogenase, ribosomal protein L30/L7 family protein, and ribosome production factor 2 were proposed as potential hubs.

In terms of comparisons between variety A and Q after 2 h of methylation inhibitor treatment, 1267 DEGs can be linked via known relationships (Supplementary Materials, Figure S13). Significantly, Soltu.DM.10G001170 (21 links, upregulated and encoding 30S ribosomal protein S10), Soltu.DM.08G017320 (19 links and upregulated), Soltu.DM.07G023330 (19 links, upregulated and encoding 50S ribosomal protein L13), Soltu.DM.09G007410 (18 links, upregulated and encoding 60S ribosomal protein L23a-like), Soltu.DM.01G025520 (18 links, downregulated and encoding 40S ribosomal protein S11), Soltu.DM.12G007300 (17 links, upregulated and encoding ATP synthase delta chain), and Soltu.DM.12G024350 (17 links, downregulated and encoding the ribosome biogenesis regulatory protein) were proposed as potentially important hubs. 

In terms of comparisons between variety A and Q after 6 h of methylation inhibitor treatment, 885 DEGs can be linked via known relationships (Supplementary Materials, Figure S14). Significantly, Soltu.DM.08G018700 (18 links and upregulated), Soltu.DM.01G025520 (15 links and downregulated), Soltu.DM.10G024790 (14 links, upregulated), and Soltu.DM.03G005430 (14 links, downregulated) were proposed as the important hubs. 

In terms of comparisons between variety A and Q after 12 h of methylation inhibitor treatment, 810 DEGs can be linked via known relationships (Supplementary Materials, Figure S15). Soltu.DM.02G002360 (13 links, upregulated and encoding 40S ribosomal protein S28), Soltu.DM.03G005430 (12 links, downregulated), and Soltu.DM.09G007410 (11 links, upregulated) were proposed as potentially important hubs. 

In terms of comparisons between variety A and Q after 2 h of simulated drought treatment, 368 DEGs can be linked via known relationships (Supplementary Materials, Figure S16). Significantly, Soltu.DM.04G004700 (five links, downregulated, and encoding ribosomal L1 domain-containing protein 1), Soltu.DM.07G003850 (four links, upregulated, and encoding 4-coumarate--CoA ligase 1), Soltu.DM.05G014770 (three links, downregulated, and encoding the DNA-directed RNA polymerase beta subunit), Soltu.DM.06G023000 (three links, downregulated, and encoding hexokinase), and Soltu.DM.12G024900 (three links and upregulated) were proposed as the key hubs. In terms of comparisons between variety A and Q after 6 h of simulated drought treatment, 749 DEGs can be linked via known relationships (Supplementary Materials, Figure S16b). Significantly, Soltu.DM.12G007300 (15 links and upregulated), Soltu.DM.05G008540 (12 links and upregulated), Soltu.DM.03G025720 (11 links and upregulated), Soltu.DM.01G025520 (11 links and downregulated), Soltu.DM.09G007410 (10 links and upregulated), Soltu.DM.01G000880 (10 links and upregulated), and Soltu.DM.12G009760 (10 links and upregulated) were proposed as potential hubs. In terms of comparisons between variety A and Q after 12 h of simulated drought treatment, 340 DEGs can be linked via known relationships (Supplementary Materials, Figure S17). Significantly, Soltu.DM.10G024790 (10 links and upregulated), Soltu.DM.01G025520 (10 links and downregulated), Soltu.DM.07G003860 (10 links and downregulated), and Soltu.DM.12G009760 (9 links and upregulated) were proposed as potential hubs.

In terms of comparisons between variety A and Q after 2 h of dual treatment, 3003 DEGs can be linked via known relationships (Figure 7). Significantly, Soltu.DM.07G000030 (57 links, upregulated, and encoding an unknown protein involved in defense mechanisms), Soltu.DM.08G021990 (54 links, downregulated, and encoding phosphoribulokinase), Soltu.DM.01G049950 (50 links, upregulated, and encoding DNA replication licensing factor MCM4), Soltu.DM.01G024810 (51 links, upregulated, and encoding DNA replication licensing factor MCM7), Soltu.DM.02G013800 (47 links, upregulated, and encoding DNA replication licensing factor MCM3), Soltu.DM.08G001360 (40 links, downregulated, and encoding photosystem I reaction center subunit psaK), Soltu.DM.11G004640 (39 links, downregulated, and encoding malate dehydrogenase (NADP)), Soltu.DM.11G015130 (40 links, downregulated, and encoding DNA replication licensing factor MCM2), and Soltu.DM.10G019980 (37 links, downregulated, and encoding Fructose-1,6-bisphosphatase) were proposed as potentially important hubs.

In terms of comparisons between variety A and Q after 6 h of dual treatment, 2839 DEGs can be linked via known relationships (Figure 8). Significantly, Soltu.DM.12G007300 (72 links, upregulated), Soltu.DM.02G032830 (66 links, upregulated, and encoding peptidyl-prolyl cis-trans isomerase CYP38), Soltu.DM.07G013280 (63 links, upregulated, and encoding peroxiredoxin Q), Soltu.DM.03G032490 (55 links, upregulated, and encoding magnesium protoporphyrin IX methyltransferase), Soltu.DM.07G023330 (52 links, upregulated), Soltu.DM.04G034750 (51 links, upregulated), Soltu.DM.07G023020 (51 links, upregulated, unknown protein), Soltu.DM.05G010760 (53 links, upregulated, and encoding mitotic spindle checkpoint protein MAD2), Soltu.DM.11G020850 (50 links, upregulated, and encoding 50S ribosomal protein L9), Soltu.DM.08G001360 (51 links, upregulated), and Soltu.DM.08G030260 (50 links, upregulated, and encoding 50S ribosomal protein L11) were proposed as potentially important hubs.

In terms of comparisons between variety A and Q after 12 h of dual treatment, 1752 DEGs can be linked via known relationships (Figure 9). Significantly, Soltu.DM.07G000030 (39 links and upregulated), Soltu.DM.01G024810 (33 links and upregulated), Soltu.DM.01G049950 (33 links and upregulated), Soltu.DM.02G013800 (33 links and upregulated), Soltu.DM.01G025520 (14 links and downregulated), Soltu.DM.12G009760 (11 links and upregulated), Soltu.DM.07G021850 (15 links, downregulated, and encoding citrate synthase), and Soltu.DM.12G030010 (11 links, downregulated, and encoding malate dehydrogenase) were proposed as potential hubs. 

### 3.4. Analysis of Methylation Sites in the Promoter of Key DEGs

**Figure 10:**
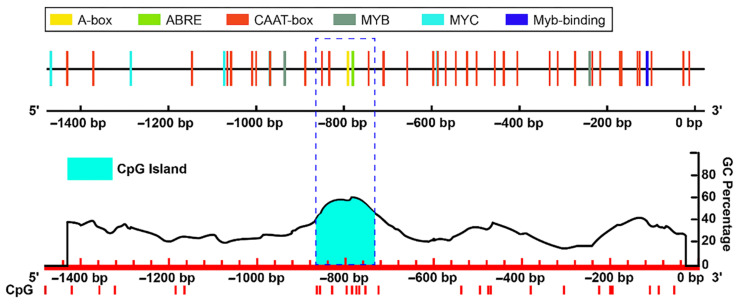
Analysis of glutathione S-transferase gene promoter.

CpG island prediction and cis-element analysis were performed on the promoter of DEGs enriched by KEGG pathways related to plant drought resistance. The results (Figure 10) showed that Soltu.DM.09G006730 related to the plant glutathione metabolism pathway was predicted, and its CpG island was located between −736and −866 in the promoter sequence of this gene, with a size of 131 bp (Figure 10). Next, we used the online database Plant Care to analyze the cis action element of the gene promoter sequence. As shown in Figure 10, there were ABRE, CAAT-box, and A-box elements in the CpG island region of the Soltu.DM.09G006730 gene, of which ABRE and CAAT -box are related to the response of plants to drought stress. The above results indicate that when potatoes respond to drought stress, the ABRE and CAAT-box elements in the promoter region of the glutathione transferase gene activate the expression of the gene through DNA demethylation to cope with stress.

### 3.5. Verification of qPCR

Twenty genes were randomly selected from DEGs, and qPCR was used to detect the relative expression of these genes in Atlantic and Qingshu 9 under different treatments (Supplementary Materials, Figure S18). The results show that the qPCR results and the transcriptome sequencing results have basically the same change trends. This demonstrates the reliability of the transcriptome sequencing data and further validates the response of these genes to drought and DNA methylation inhibitors.

## 4. Discussion

Various epigenetic modifications before transcription, such as DNA methylation, histone, and chromatin remodeling modifications, also play an important role in regulating plants’ response to drought stress [4,5]. Under drought-stress conditions, plants start or stop a series of cascade reactions (response pathways) caused by genes related to stress response through methylation and demethylation of genomic DNA and increase the activity of enzymes in response to stress, thereby resisting drought, as stress is known to impact plant growth and development [35]. Therefore, understanding the dynamic changes in plant epigenetic DNA methylation modification under drought stress will help to study functional gene expression and further clarify the molecular mechanisms of plants’ responses to drought. This result also fully implies epigenetic modifications such as DNA methylation. The potential importance and application value of our research are in the fields of plant drought resistance and breeding. Epigenetic modification with DNA methylation as the core is involved in the balance of gene expression, genome stability, and adaptability, which is very important for improving crop traits [38]. At present, there is little research on the relationship between potato drought resistance and DNA methylation. In a previous study, the responses of different potato cultivars to mannitol and 5-azadC were similar. With treatments of mannitol or 5-azadC, the dry/fresh weight, shoot height, leaf number, and chlorophyll content decreased significantly, while the activities of SOD, POD, CAT, and contents of proline and MDA increased significantly. However, the number of branches, root length, and average root thickness did not change significantly, indicating that the regulatory pathways may be different [39]. 

Analysis of DNA methylation polymorphism in response to drought stress in rice has shown that drought-sensitive genotypes are more likely to have hypermethylation than drought-tolerant genotypes [11]. In this study, a large batch of RNA-seq was used to further study the effect of methylation between different varieties on the drought stress response. This study found that under untreated conditions, there were 1295 DEGs between the two varieties (Figure 1e). Through functional annotation analysis, we found that these differential genes mainly belong to plant hormone signal transduction, defense response, plant–pathogen interaction, protein processing in the endoplasmic reticulum, and starch and sucrose metabolism (Supplementary Materials, Figure S5). 

In both drought-tolerant variety Q and drought-sensitive variety A, many genes were upregulated or downregulated after 6 h of drought stress (Figure 1B), which lasted for up to 12 h. Compared with drought-sensitive variety A, the number of DEGs in drought-tolerant variety Q decreased sharply after 12 h of drought treatment, which indicated that variety Q could quickly adapt to drought stress by regulating gene expression. At each treatment time point under the dual-treatment condition (Figure 1c), the number of DEGs in variety Q was lower than that in variety A, and the number of highly expressed genes in variety Q was lower than that in variety A regardless of drought or dual-treatment conditions. A (Figure 1d). These results also indicated that variety Q only needs to change relatively few gene expressions to cope with drought stress. 

After 2 h of methylation inhibitor treatment, many genes began to be upregulated or downregulated (Figure 1a). In drought-sensitive variety A, the difference between treatment for 6 h and treatment for 2 h was not significant, and the difference between treatment for 6 h and treatment for 12 h was not obvious, indicating that treatment for 2 h can inhibit or induce gene expression. In drought-tolerant variety Q, the difference between treatment for 6 h and treatment for 2 h was larger, and there were 783 DEGs between treatment for 6 h and treatment for 12 h, indicating that the methylation inhibitor could continue to act on variety Q. These results indicate that, to some extent, the background methylation complexity of the genomic DNA of variety Q may be higher than that of variety A. 

We can also infer the same result from the Venn diagram (Supplementary Materials, Figures S3 and S4). In cultivar A (Figure 2a), the number of specific DEGs under dual treatment conditions is always much greater than that of methylation inhibitors and drought treatments. For variety Q, the number of DEGs unique to the dual treatment dropped sharply when the drought treatment was 6 h. Regardless of variety A or variety Q, the number of DEGs shared by drought treatment and methylation inhibitors was far more than that of unique DEGs after 2 h of treatment, which indicated that potato genome DNA methylation was deeply involved in the drought stress response. In addition, under the conditions of methylation inhibition and drought treatment for 2 h (Figure 3a,b), the number of DEGs unique to drought-tolerant variety Q is more than that of drought-sensitive variety A, which also indicates that the level of DNA methylation in variety Q is higher, and the reaction is more sensitive and faster. However, under the dual-treatment conditions, the number of DEGs specific to drought-tolerant variety Q was slightly less than that of drought-sensitive variety A, which indicated that there were certain differences in the regulation of methylation levels between the two varieties. 

According to the functional annotation enrichment analysis of DEGs, in drought-sensitive variety A (Supplementary Materials, Figure S6), under the conditions of methylation inhibitor treatment, light stress response, photosynthesis, and other pathways were first affected (2 h), followed by transcription regulation and calcium signal transduction, etc. (6 h). Under drought conditions, DNA transcriptional regulation and other pathways first reacted in large numbers (2 h), and after 6 h of treatment, genes in the photosynthesis pathway began to be differentially expressed in large numbers. The situation is different in drought-tolerant variety Q (Supplementary Materials, Figure S7). Under methylation inhibitors and drought treatment, they react quickly (2 h) in the pathways of transcription regulation and calcium signal transduction, but at the same time, under treatment with methylation inhibitors, membrane system components are also regulated. As the treatment time increases, the metabolic pathways related to photosynthesis are gradually affected. 

In our previous work, 5-azadC was used to treat different drought-sensitive potato varieties [34] and we found that DNA demethylation under drought stress is involved in regulating the phenotypic traits and physiological and biochemical responses of potatoes. Similar results were also observed by application of DNA methylation inhibitor 5-azacytidine on watermelon (*Citrullus lanatus*), especially in those involved in the regulation of inositol-related genes [40], providing a preliminary insight into the altered methylation levels of watermelon cells in response to osmotic stress. Recent research suggested that wild mungbean 61 was more resistant to drought stress, with more hypermethylated DMRs and less hypomethylated DMRs after drought stress, corresponding to more downregulated DEGs than upregulated DEGs, which proved that the differentially methylated regions (DMRs) in the gene body were significantly negatively correlated with DEGs [41]. In this study, the functional annotation of DEGs between the two varieties (Figure 4) also verified this phenomenon. Under treatment with methylation inhibitors, DEGs were first significantly enriched in photosynthesis, and under drought conditions, DEGs were first significantly enriched in defense reactions and signal transduction. Therefore, these results indicate that the response process of different varieties to drought stress is different, and this process is affected by genome methylation.

As we learned from the results of PPI analysis (Figure 7, Figure 8 and Figure 9, Supplementary Materials, Figures S12–S17), under methylation treatment, the key regulatory pathways for the differences between the two varieties focus on protein translation and ATP synthesis. During drought treatment, in addition to protein translation, the key regulatory pathways for differences between the two varieties focused on DNA replication and transcription and lignin synthesis. Under dual-treatment conditions, differences in DNA replication were more significant between the two species, and the MCM complex became a key regulatory node, followed by photosynthesis, protein translation, defense response, and secondary metabolism. These results indicate that the differences in the regulation of genomic DNA methylation levels between the two varieties are mainly based on the replication and transcription of genomic DNA, protein translation, photosynthesis, and secondary metabolism.

## 5. Conclusions

Our research shows that compared with drought-sensitive variety A, drought-tolerant variety Q is more sensitive and milder in response to drought stress. Potato genome DNA methylation is deeply involved in the drought stress response. The DNA methylation levels between the two varieties are significantly different in DNA replication and transcription, protein translation, photosynthesis, defense response, and secondary metabolism regulation. Variety Q’s complexity of background methylation of genomic DNA may be higher than that of variety A, and methylation regulation is more refined. More importantly, we also identify some hub proteins that could be used as key candidate genes in molecular breeding.

## Data Availability

Data supporting reported results can be found in the NCBI database with the BioProject number PRJNA783478.

## Supplementary Materials

The following are available online at https://www.mdpi.com/article/10.3390/genes13122260/s1, Supplementary Figure S1. The PCA (principal component analysis) results of testing the repeatability of samples in different treatment groups for varieties A (a) and Q (b). Supplementary Figure S2. Heat map of variety A and Q genes responding to three kinds of treatment at the transcription level. To be considered differentially expressed, a transcript must have FPKM ≥ 0.3 in at least one sample, a two-fold (or greater change) between samples, and p ≤ 0.05. Blue indicates high expression, white indicates intermediate expression, and red indicates low expression. Supplementary Figure S3. Heat map of variety A genes responding to three kinds of treatment at the transcription level. Red indicates high expression, white indicates intermediate expression, and green indicates low expression. Supplementary Figure S4. Heat map of variety Q genes responding to three kinds of treatment at the transcription level. Red indicates high expression, white indicates intermediate expression, and green indicates low expression. Supplementary Figure S5. Statistics and functional enrichment of DEGs between varieties A and Q under control conditions. (a) Heat map of DEGs. Blue indicates high expression, white indicates intermediate expression, and red indicates low expression. (b) Histogram of most enriched GO classifications. (c) Histogram of most enriched KEGG pathway. (d) Histogram of the most significant enriched KEGG pathway. Supplementary Figure S6. Histogram of most enriched GO classifications of DEGs in variety A under treatments. Supplementary Figure S7. Histogram of most enriched GO classifications of DEGs in variety Q under treatments. Supplementary Figure S8. Histogram of most enriched KEGG pathway of DEGs in variety A under treatments. Supplementary Figure S9. Histogram of most enriched KEGG pathway of DEGs in variety Q under treatments. Supplementary Figure S10. Histogram of the most significantly enriched KEGG pathway of DEGs in variety A under treatments. Supplementary Figure S11. Histogram of the most significantly enriched KEGG pathway of DEGs in variety Q under treatments. Supplementary Figure S12. The PPI network of DEGs from variety A versus Q under control conditions. Supplementary Figure S13. The PPI network of DEGs from variety A versus Q under methylation inhibitor treatment for 2 h. Supplementary Figure S14. The PPI network of DEGs from variety A versus Q under treatment for 6 h. (a) Methylation inhibitor treatment. (b) Simulated drought treatment. Supplementary Figure S15. The PPI network of DEGs from variety A versus Q under methylation inhibitor treatment for 12 h. Supplementary Figure S16. The PPI network of DEGs from variety A versus Q under simulated drought treatment for 2 h. Supplementary Figure S17. The PPI network of DEGs from variety A versus Q under simulated drought treatment for 12 h. Supplementary Figure S18. qPCR validation of DEGs under drought and dual treatment. Supplementary Table S1. Primers list of selected DEGs for qPCR validation. Supplementary Table S2. Assessment of the RNA-seq data quality. Supplementary Table S3. The FPKM value of identified genes in all samples. Supplementary Table S4. Functional annotation for identified genes in this study. Supplementary Table S5. Statistics of functional annotation of all identified genes in this study. Supplementary Table S6. FPKM values for DEGs in all samples. Supplementary Table S7. The FPKM value and function annotation for the DEGs in variety A of comparison under three kinds of treatments. Supplementary Table S8. The FPKM value and function annotation for the DEGs in variety Q, comparing three kinds of treatments. Supplementary Table S9. The FPKM value and function annotation for the DEGs between varieties A and Q, comparing three kinds of treatments.

## References

[1] Golldack D., Li C., Mohan H., Probst N. (2014). Tolerance to drought and salt stress in plants: Unraveling the signaling networks. Front. Plant Sci..

[2] Shinozaki K., Yamaguchi-shinozaki K., Seki M. (2003). Regulatory network of gene expression in the drought and cold stress responses. Cur. Opin. Plant Biol..

[3] Rabbani M.A., Maruyama K., Abe H., Khan M.A., Katsura K., Ito Y., Yoshiwara K., Seki M., Shinozaki K., Yamaguchi-Shinozaki K. (2003). Monitoring expression profiles of rice genes under cold, drought, and high-salinity stresses and abscisic acid application using cDNA microarray and RNA gel-blot analyses. Plant Physiol..

[4] Gallusci P., Hodgman C., Teyssier E., Seymour G.B. (2016). DNA Methylation and Chromatin Regulation during Fleshy Fruit Development and Ripening. Front. Plant Sci..

[5] Pikaard C.S., Mittelsten S.O. (2014). Epigenetic regulation in plants. Cold Spring Harb. Perspect. Biol..

[6] Vanyushin B.F., Ashapkin V.V. (2011). DNA methylation in higher plants: Past, present and future. BBA-Gene Struct. Expr..

[7] Zhao Y.L., Ye W.W., Wang J.J., Fan B.X., Song L.Y. (2009). Review of DNA Methylation and Plant Stress-Tolerance. Acta Bot. Boreal.-Occident. Sin..

[8] Zhang P., Wang J., Geng Y., Dai J.R., Zhong Y., Chen Z.Z., Zhu K., Wang X.Z., Chen S.Y. (2015). MSAP-based analysis of DNA methylation diversity in tobacco exposed to different environments and at different development phases. Bioch. Syst. Ecol..

[9] Kaur A., Grewal A., Sharma P. (2018). Comparative analysis of DNA methylation changes in two contrasting wheat genotypes under water deficit. Biol. Plant..

[10] Liang D., Zhang Z., Wu H., Huang C., Shuai P., Ye C., Tang S., Wang Y., Yang L., Wang J. (2014). Single-base-resolution methylomes of *Populus trichocarpa* reveal the association between DNA methylation and drought stress. BMC Genet..

[11] Joel A.J. (2013). Epigenetic responses to drought stress in rice (*Oryza sativa* L.). Physiol. Mol. Biol. Plants.

[12] Garg R., Narayana C.V., Shankar R., Jain M. (2015). Divergent DNA methylation patterns associated with gene expression in rice cultivars with contrasting drought and salinity stress response. Sci. Rep..

[13] Chwialkowska K., Nowakowska U., Mroziewicz A., Szarejko I., Kwasniewski M. (2016). Water-deficiency conditions differently modulate the methylome of roots and leaves in barley (*Hordeum vulgare* L.). J. Exp. Bot..

[14] Liu J., Zhang X.Y., Deng X. (2018). Analysis of DNA methylation of *Boea hygrometrica* under dehydration stress based on methylation sensitive amplification polymorphism. J. Fujian Agric. For. Univ. (Nat. Sci. Edit.).

[15] Tang X.M., Tao X., Wang Y., Ma D.-W., Li D., Yang H., Ma X.-R. (2014). Analysis of DNA methylation of perennial ryegrass under drought using the methylation-sensitive amplification polymorphism (MSAP) technique. Mol. Genet. Genom..

[16] Bhardwaj J., Mahajan M., Yadav S.K. (2013). Comparative analysis of DNA methylation polymorphism in drought sensitive (HPKC2) and tolerant (HPK4) genotypes of horse Gram (*Macrotyloma uniflorum*). Biochem. Genet..

[17] Guo H.X., Chen L., Lou Q.J.X., Hui L.S., Luo L.J. (2014). Changes in DNA methylation pattern in a water-saving and drought-resistance rice variety at three leaf and four-leaf stages after drought domestication Z. Chin. J. Rice Sci..

[18] Meng D.W., Wang Y., Li P.X., Zhao Y.W., Zhou Y., Han Y., Lang C.J., Jin T.C., Yang L.P. (2020). Drought-introduced DNA demethylation of *AtGSTF14* Gene. Mol. Plant Breed..

[19] Feng S.B. (2017). Research on DNA Methylation Variation Induced by Drought Stress in Cassava.

[20] Abid G., Mingeot D., Muhovski Y., Mergeai G., Aouida M., Abdelkarim S., Aroua I., El Ayed M., M’hamdi M., Sassi K. (2017). Analysis of DNA methylation patterns associated with drought stress response in faba bean (*Vicia faba* L.) using methylation-sensitive amplification polymorphism (MSAP). Environ. Exp. Bot..

[21] Riddle N.C., Richards E.J. (2002). The control of natural variation in cytosine methylation in Arabidopsis. Genetics.

[22] Yuan Y., Zhu S., Fang T.T., Jiang J.J., Wang Y.P. (2019). Analysis of drought resistance and DNA methylation level of resynthesized *Brassica napus*. Acta Agron. Sin..

[23] Miura K., Agetsuma M., Kitano H., Yoshimura A., Matsuoka M., Jacobsen S.E., Ashikari M. (2009). A metastable DWARF1 epigenetic mutant affecting plant stature in rice. Proc. Natl. Acad. Sci. USA.

[24] Kottler E.J., Vanwallendael A., Franks S.J. (2018). Experimental treatment with a hypomethylating agent alters life history traits and fitness in *Brassica rapa*. J. bot..

[25] Xing X.C., Suo L.G., Xiao C.Y., Liu M., Liu H., Cui J. (2017). Effect of 5-azacytidine on photosynthesis of cucumber seedlings under low temperature and light intensity. Agric. Res. Arid Areas.

[26] Xu R., Zhang W.Z., Chen L.L., Ding X., Xie Z., Lin Y., Xin J. (2017). Research progress on action mechanism of DNA demethylating agents. Drugs Clin..

[27] Mao R.T. (2014). Phylogenetics and Expression of Methyltransferases and Demethylases in Potato.

[28] Zhong L., Xu Y., Wang J. (2010). The effect of 5-azacytidine on wheat seedlings responses to NaCl stress. Biol. Plant..

[29] Bai J. (2019). Physiological Response and DNA Methylation of Brassica Napus under Low Temperature Stress.

[30] Ramírez D.A., Yactayo W., Rens L.R., Rolando J.L., Palacios S., de Mendiburu F., Mares V., Barreda C., Loayza H., Monneveux P. (2016). Defining biological thresholds associated to plant water status for monitoring water restriction effects: Stomatal conductance and photosynthesis recovery as key indicators in potato. Agric. Water Manag..

[31] Law R.D., Suttle J.C. (2003). Transient decreases in methylation at 5′-CCGG-3′ sequences in potato (*Solanum tuberosum* L.) meristem DNA during progression of tubers through dormancy precede the resumption of sprout growth. Plant Mol. Biol..

[32] Li Y.Y., Cheng P., Xiong X.Y., Hong Y.H. (2012). DNA methylation in potato under drought stress. Chin. Potato J..

[33] Wang F., Shi R., Wang J. (2013). Genetic variation of DNA methylation in potato shoot tips after the cryopreservation by vitrification approach. Mol. Plant Breed..

[34] Li P.C., Bi Z.Z., Liang W.J., Sun C., Zhang J.L., Bai J.P. (2019). DNA methylation involved in regulating drought stress response of potato. Acta Agron. Sin..

[35] Kim D., Pertea G., Trapnell C., Pimentel H., Kelley R., Salzberg S.L. (2013). TopHat2: Accurate alignment of transcriptomes in the presence of insertions, deletions and gene fusions. Genom. Biol..

[36] Schulze S.K., Kanwar R., Gölzenleuchter M., Therneau T.M., Beutler A.S. (2012). SERE: Single-parameter quality control and sample comparison for RNA-Seq. BMC Genom..

[37] McKenna A., Hanna M., Banks E., Sivachenko A., Cibulskis K., Kernytsky A., Garimella K., Altshuler D., Gabriel S., Daly M. (2010). The Genome Analysis Toolkit: A MapReduce framework for analyzing next-generation DNA sequencing data. Genom. Res..

[38] Li Y., Qian W.Q. (2017). Mechanisms of DNA methylation and demethylation in plants. Chin. Bull. Life Sci..

[39] Jiao J., Wu J., Lyu Z., Sun C., Gao L., Yan X., Cui L., Tang Z., Yan B., Jia Y. (2015). Methylation-sensitive amplified polymorphism-based genome-wide analysis of cytosine methylation profiles in *Nicotiana tabacum* cultivars. Genet. Mol. Res..

[40] Zhu F., Li M., Yan M., Qiao F., Jiang X. (2021). Integrated Transcriptome Analysis and Single-Base Resolution Methylomes of Watermelon (*Citrullus lanatus*) Reveal Epigenome Modifications in Response to Osmotic Stress. Front. Plant Sci..

[41] Zhao P., Ma B., Cai C., Xu J. (2022). Transcriptome and methylome changes in two contrasting mungbean genotypes in response to drought stress. BMC Genom..

