# Supplementary figures and images for: Transcriptomics Analysis Reveals a More Refined Regulation Mechanism of Methylation in a Drought-Tolerant Variety of Potato
Source: Genes (Basel). 2022 Nov 30;13(12):2260. doi: 10.3390/genes13122260 (PMC9778648; doi:10.3390/genes13122260)

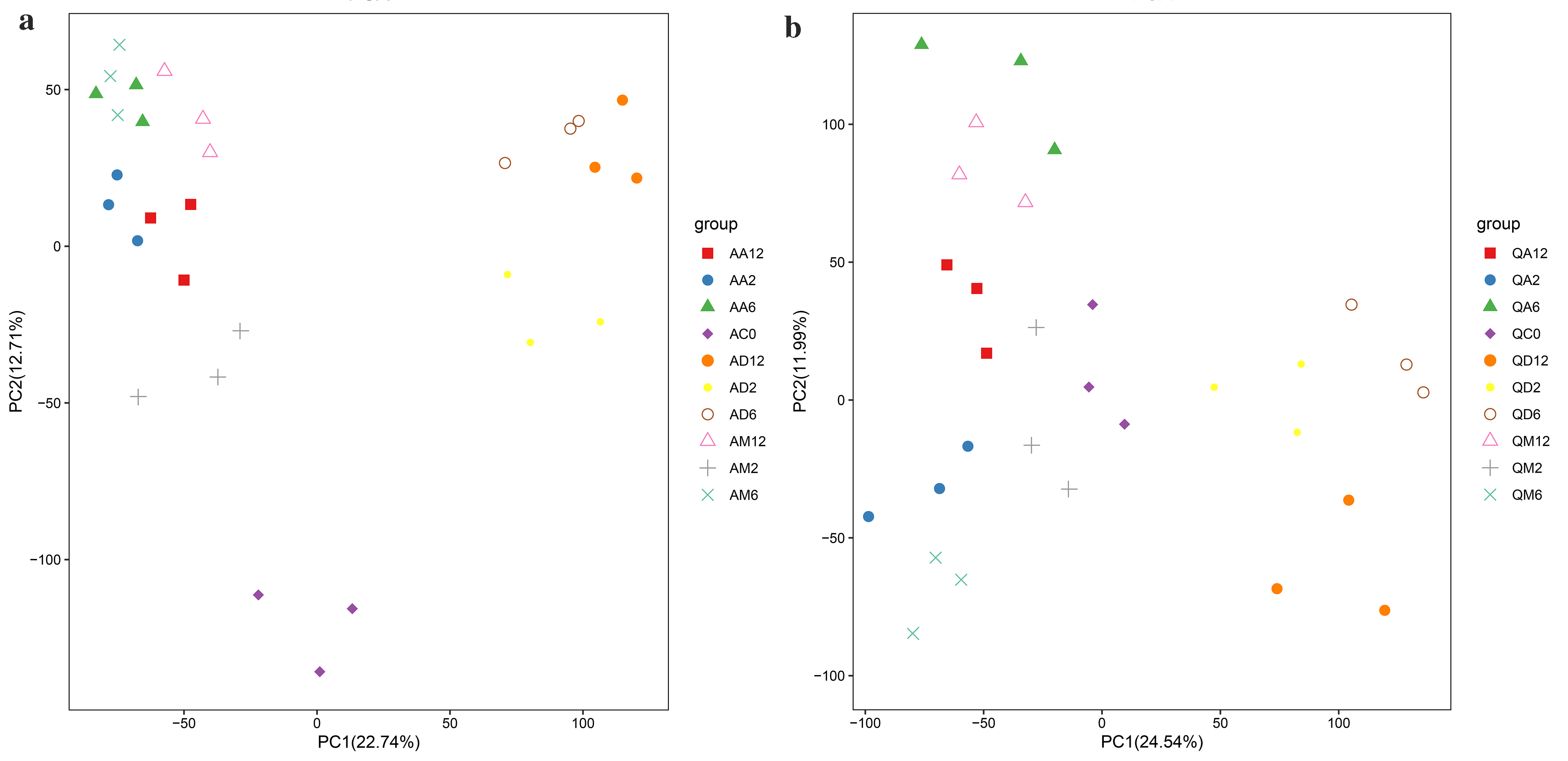

Supplement: Supplementary file 1 [file genes-13-02260-s001.zip › Supplementary Figure S1.tif]

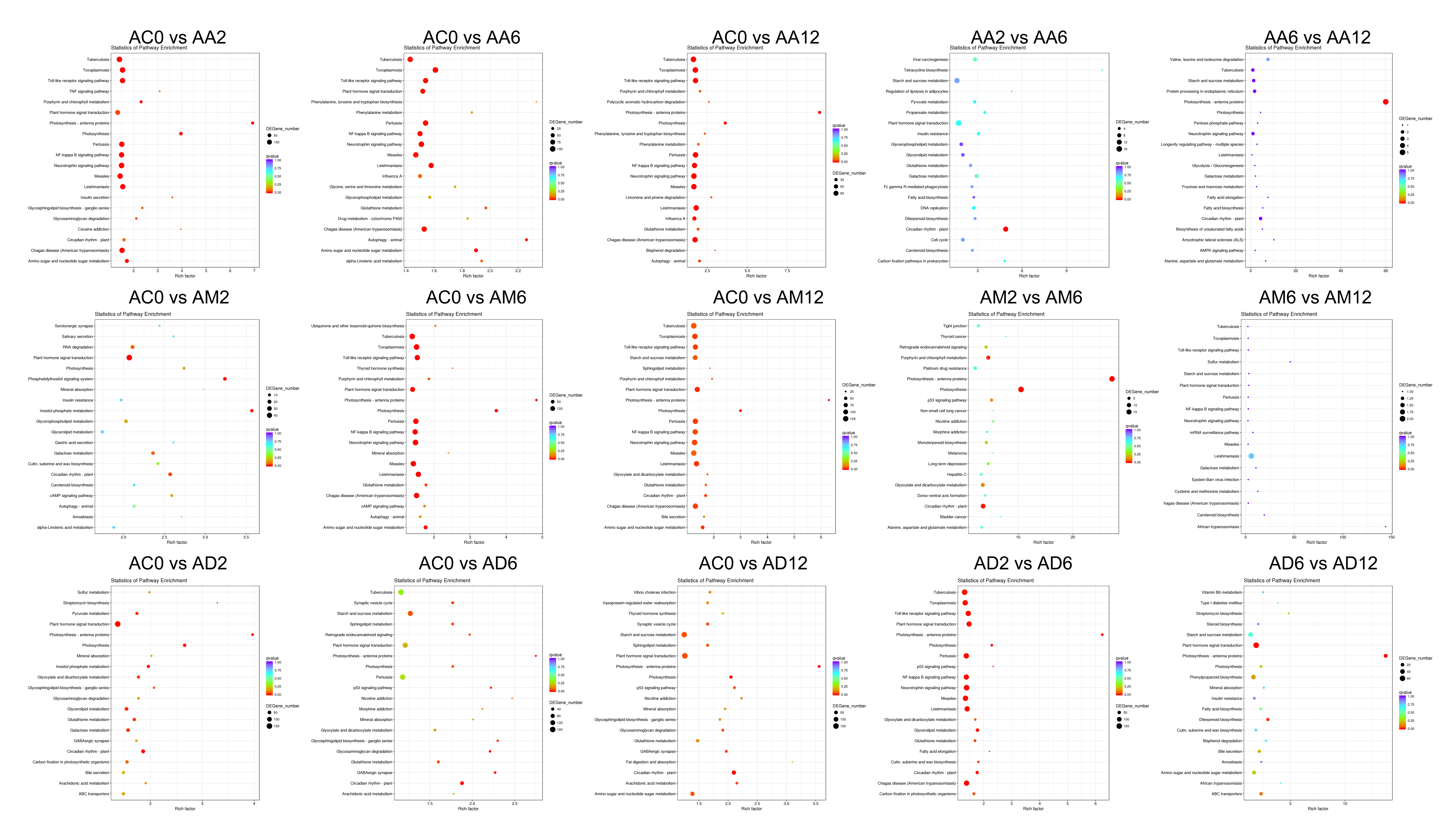

Supplement: Supplementary file 1 [file genes-13-02260-s001.zip › Supplementary Figure S10.tif]

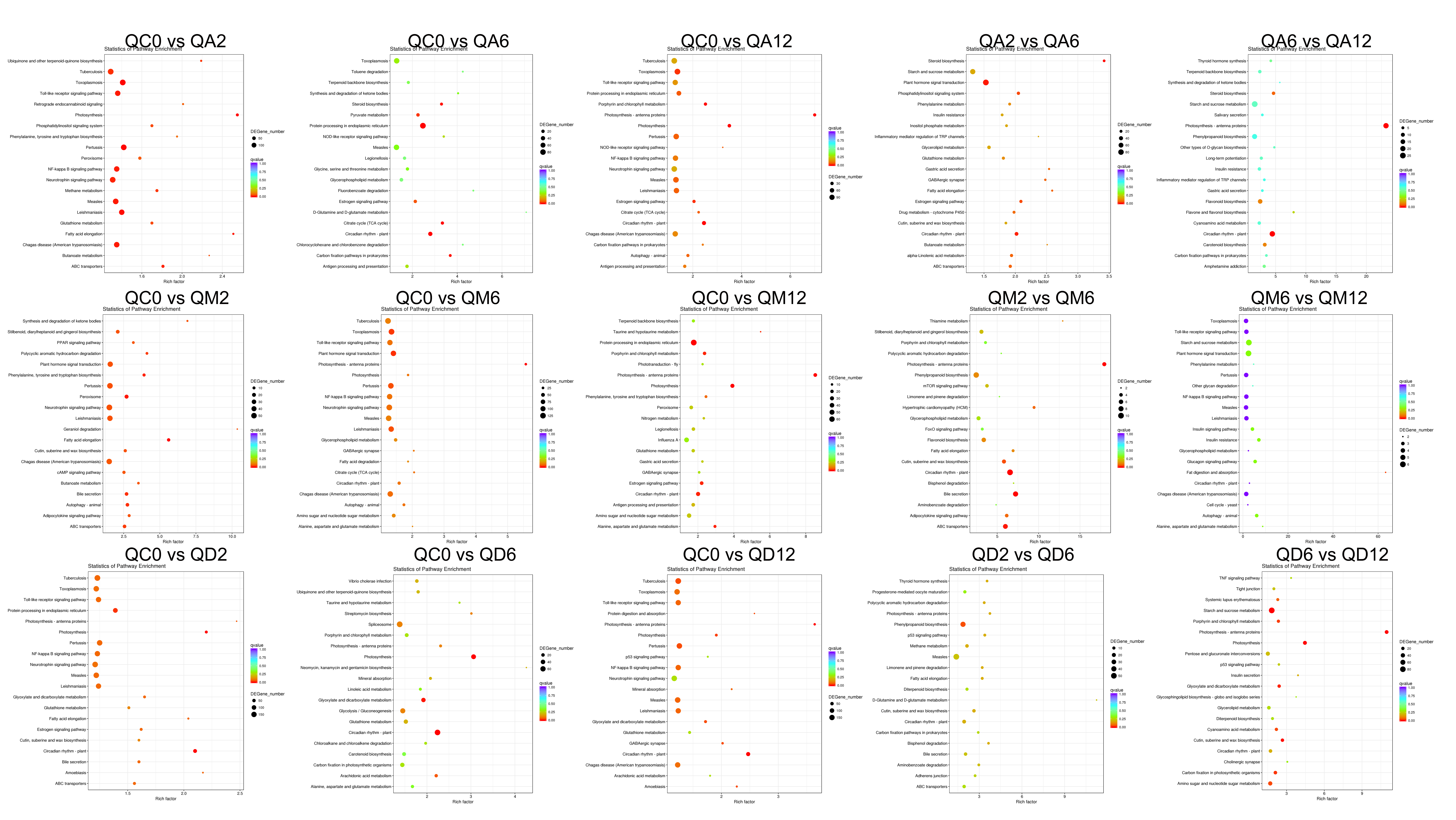

Supplement: Supplementary file 1 [file genes-13-02260-s001.zip › Supplementary Figure S11.tif]

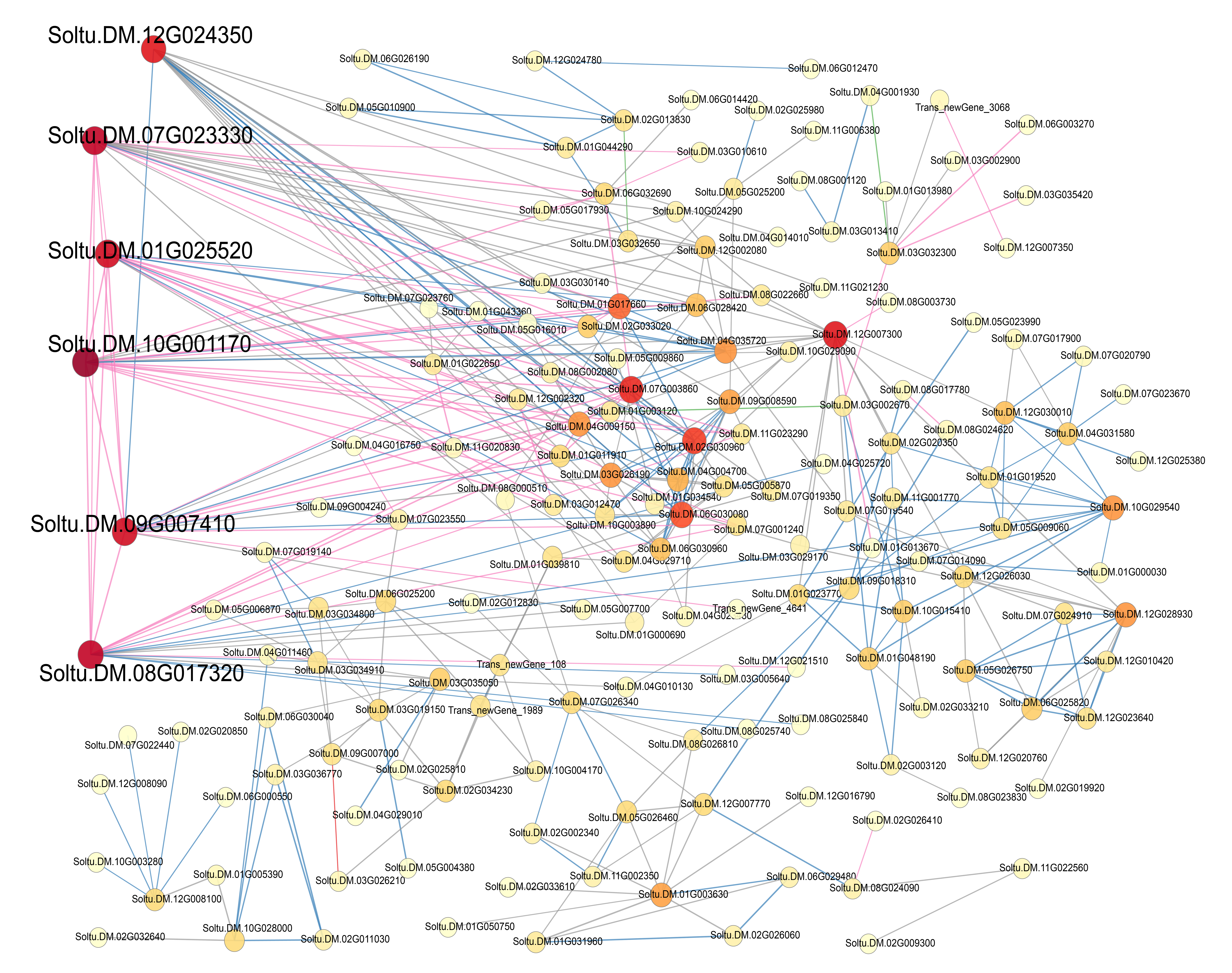

Supplement: Supplementary file 1 [file genes-13-02260-s001.zip › Supplementary Figure S13.tif]

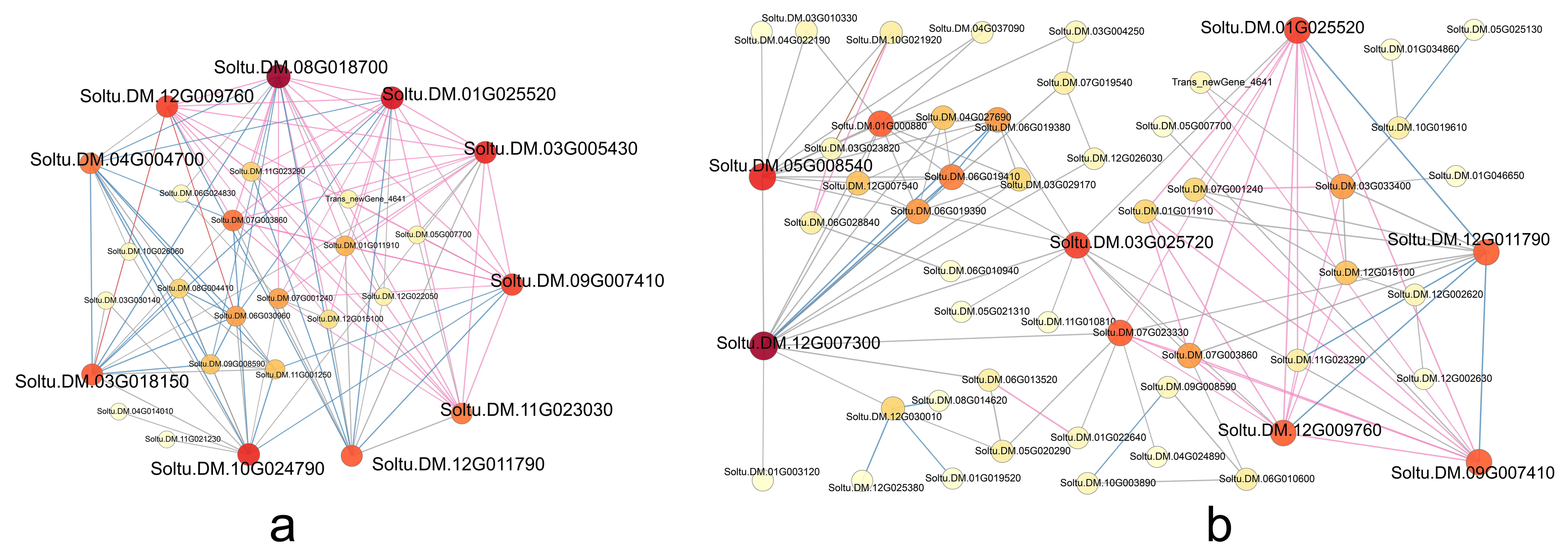

Supplement: Supplementary file 1 [file genes-13-02260-s001.zip › Supplementary Figure S14.tif]

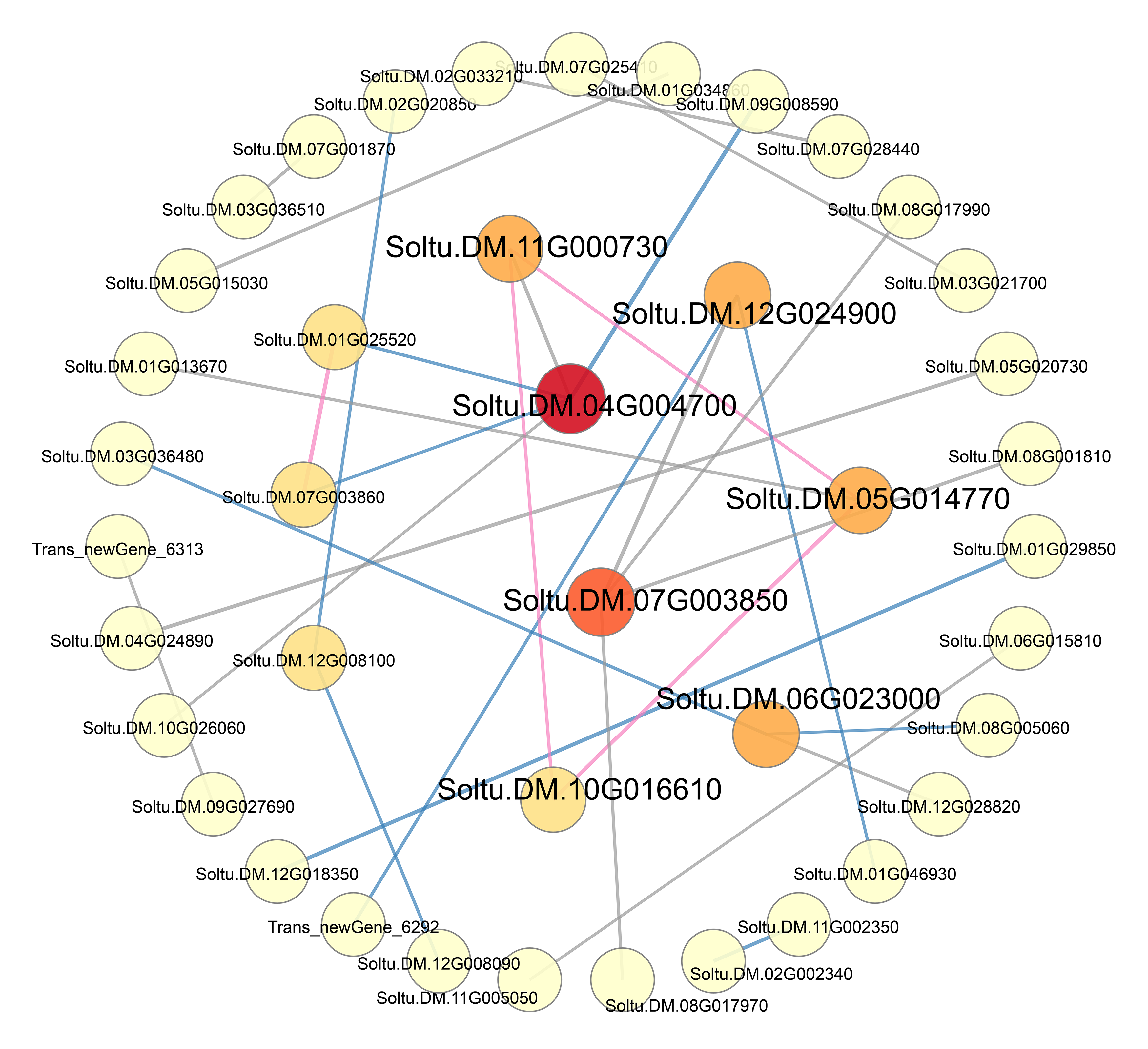

Supplement: Supplementary file 1 [file genes-13-02260-s001.zip › Supplementary Figure S16.tif]

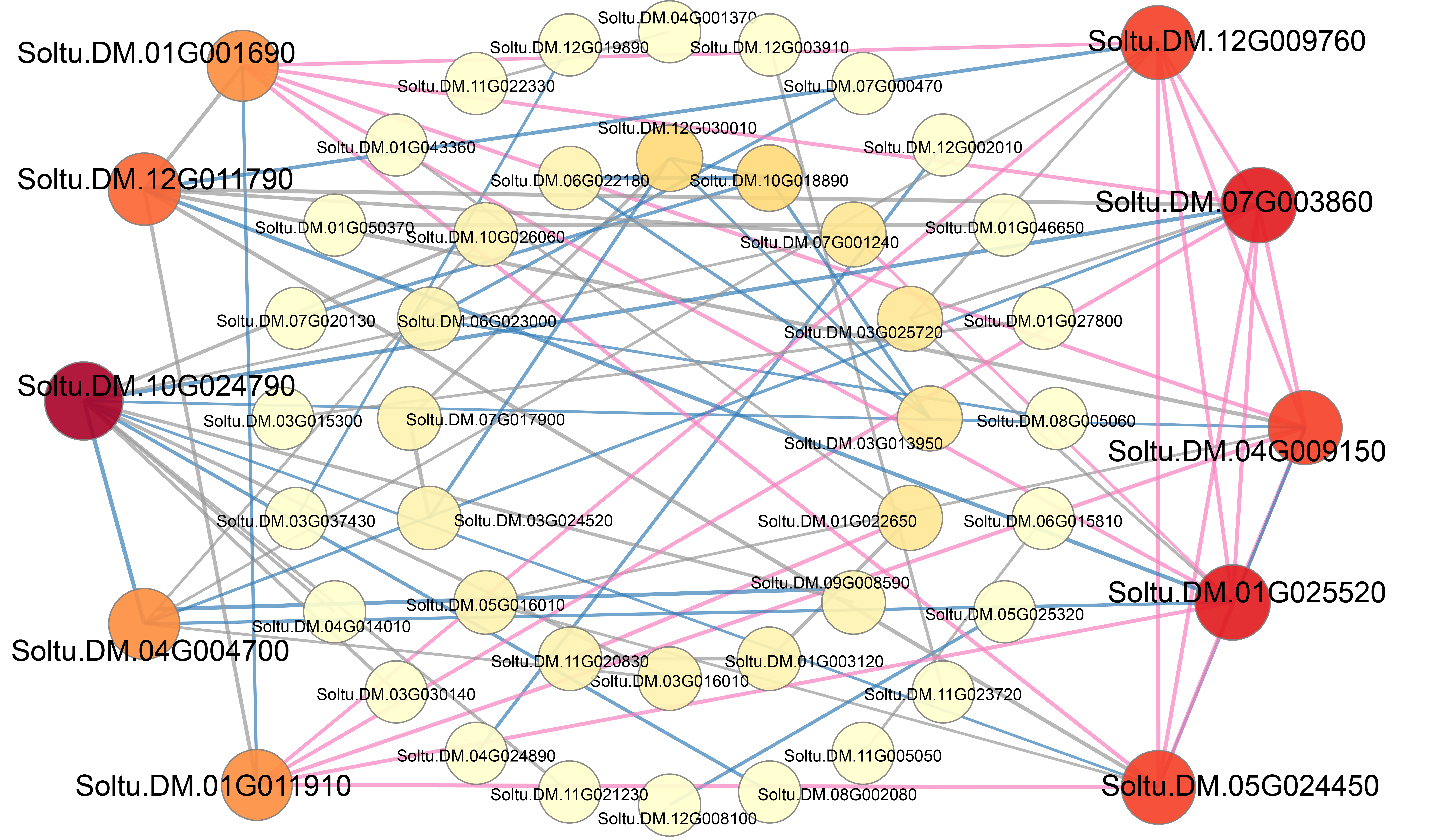

Supplement: Supplementary file 1 [file genes-13-02260-s001.zip › Supplementary Figure S17.tif]

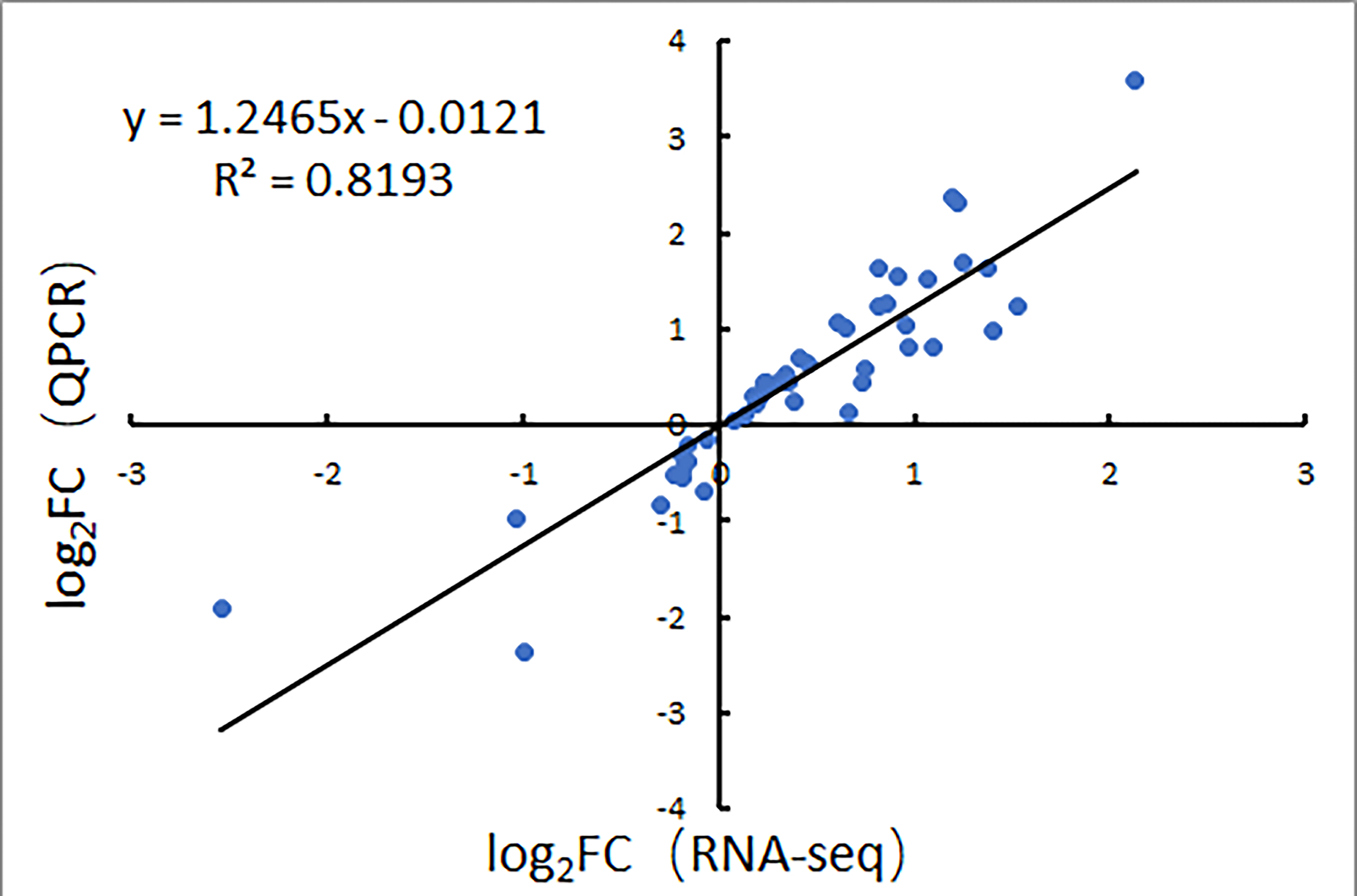

Supplement: Supplementary file 1 [file genes-13-02260-s001.zip › Supplementary Figure S18.tif]

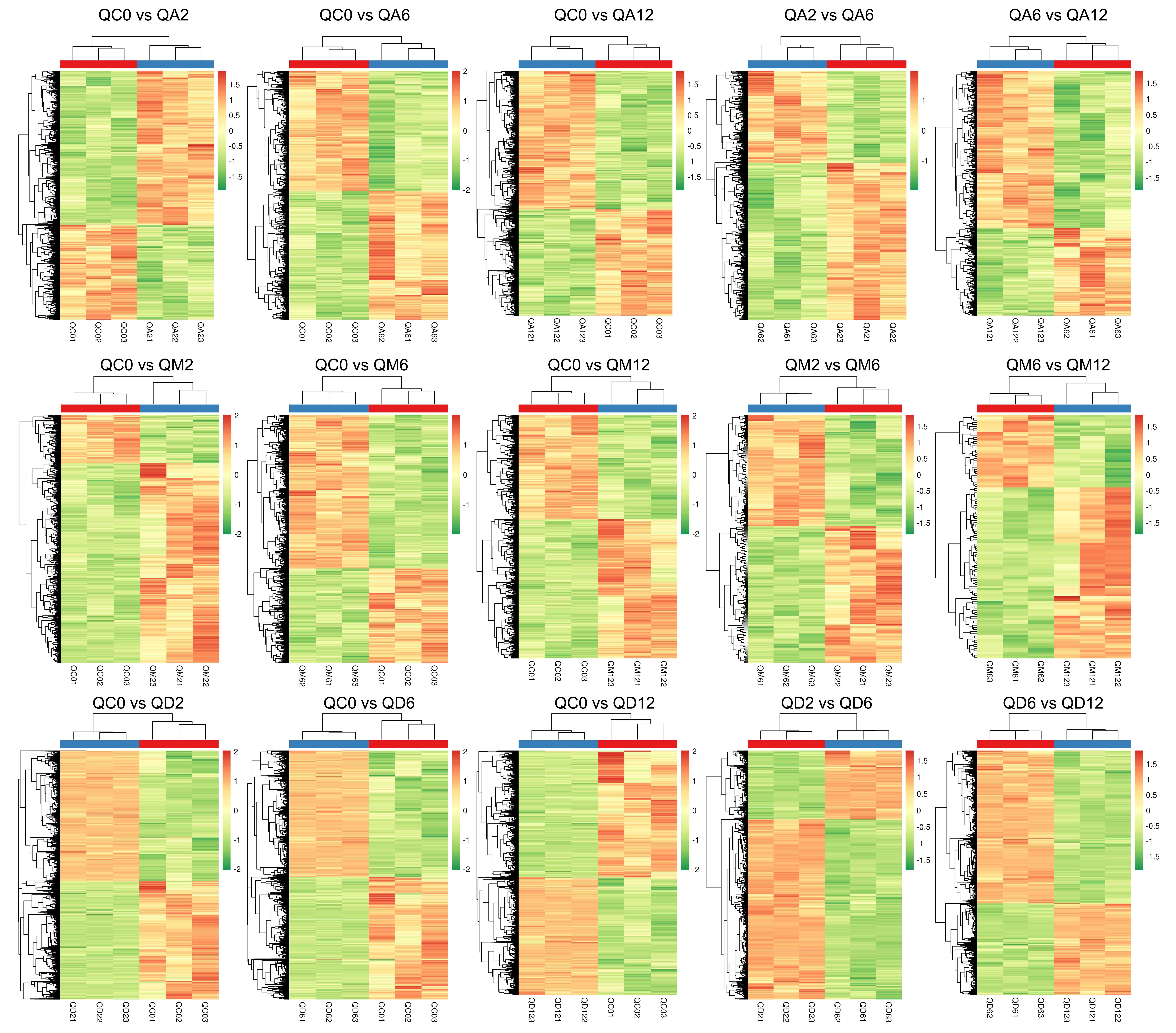

Supplement: Supplementary file 1 [file genes-13-02260-s001.zip › Supplementary Figure S4.tif]

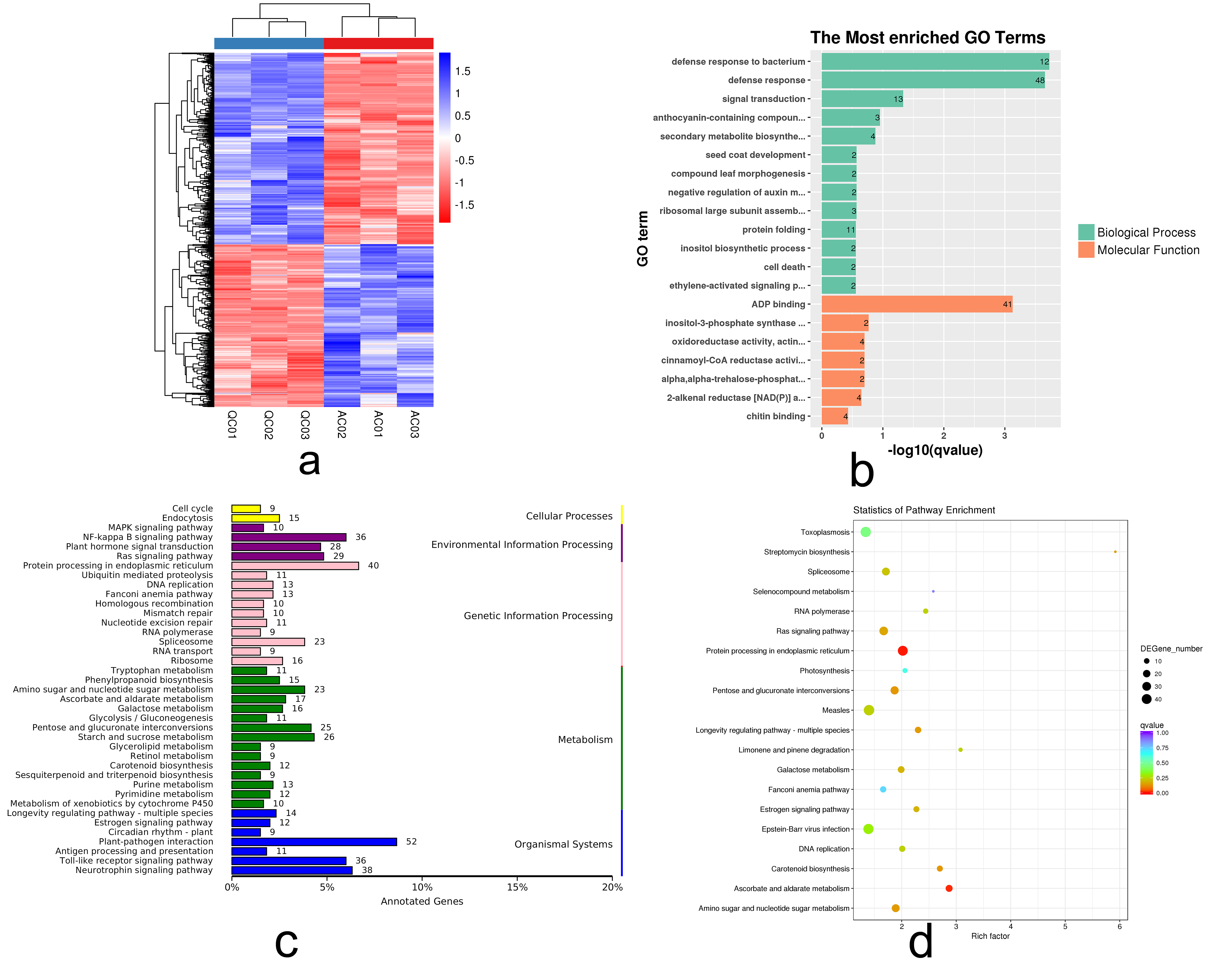

Supplement: Supplementary file 1 [file genes-13-02260-s001.zip › Supplementary Figure S5.tif]

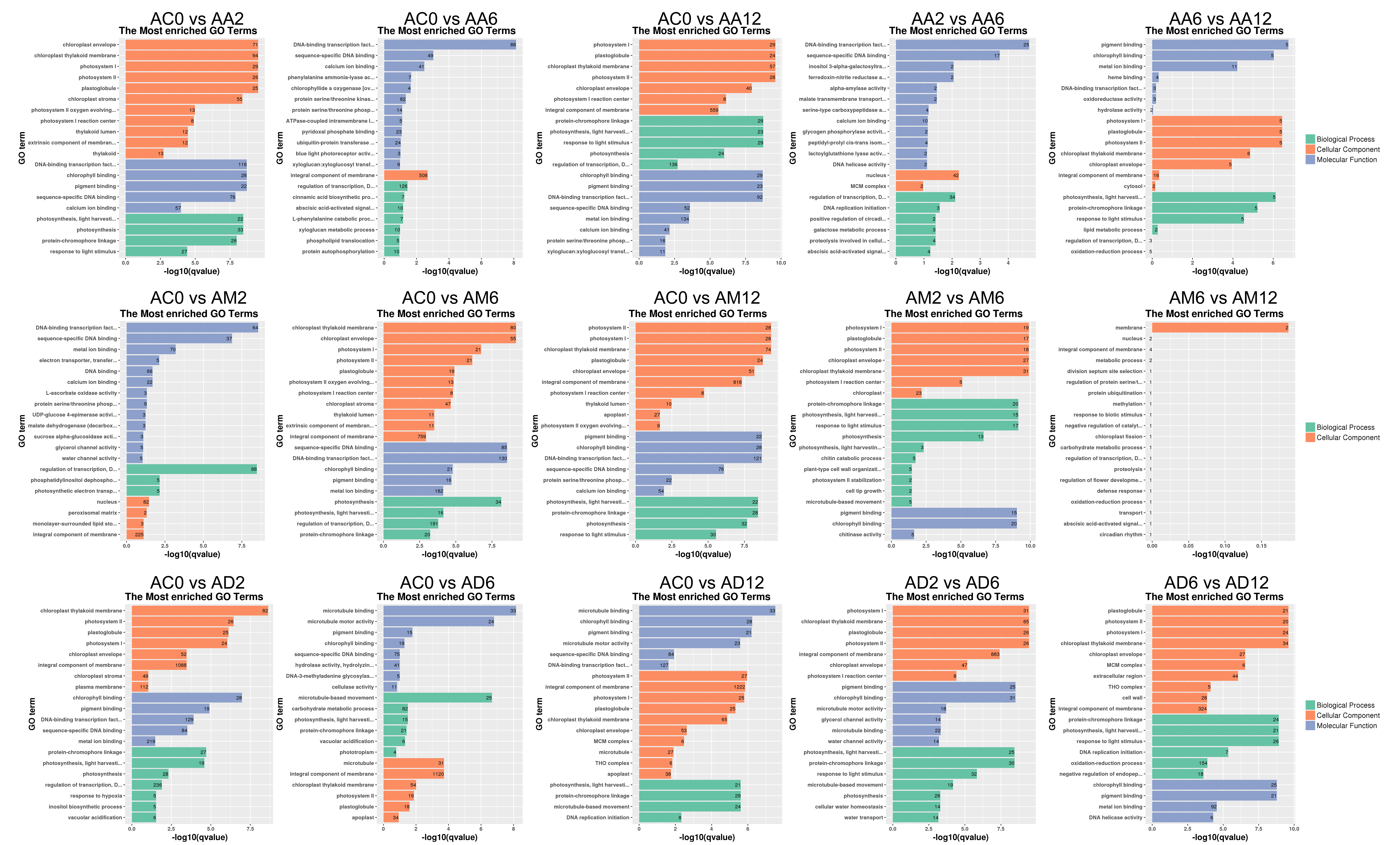

Supplement: Supplementary file 1 [file genes-13-02260-s001.zip › Supplementary Figure S6.tif]

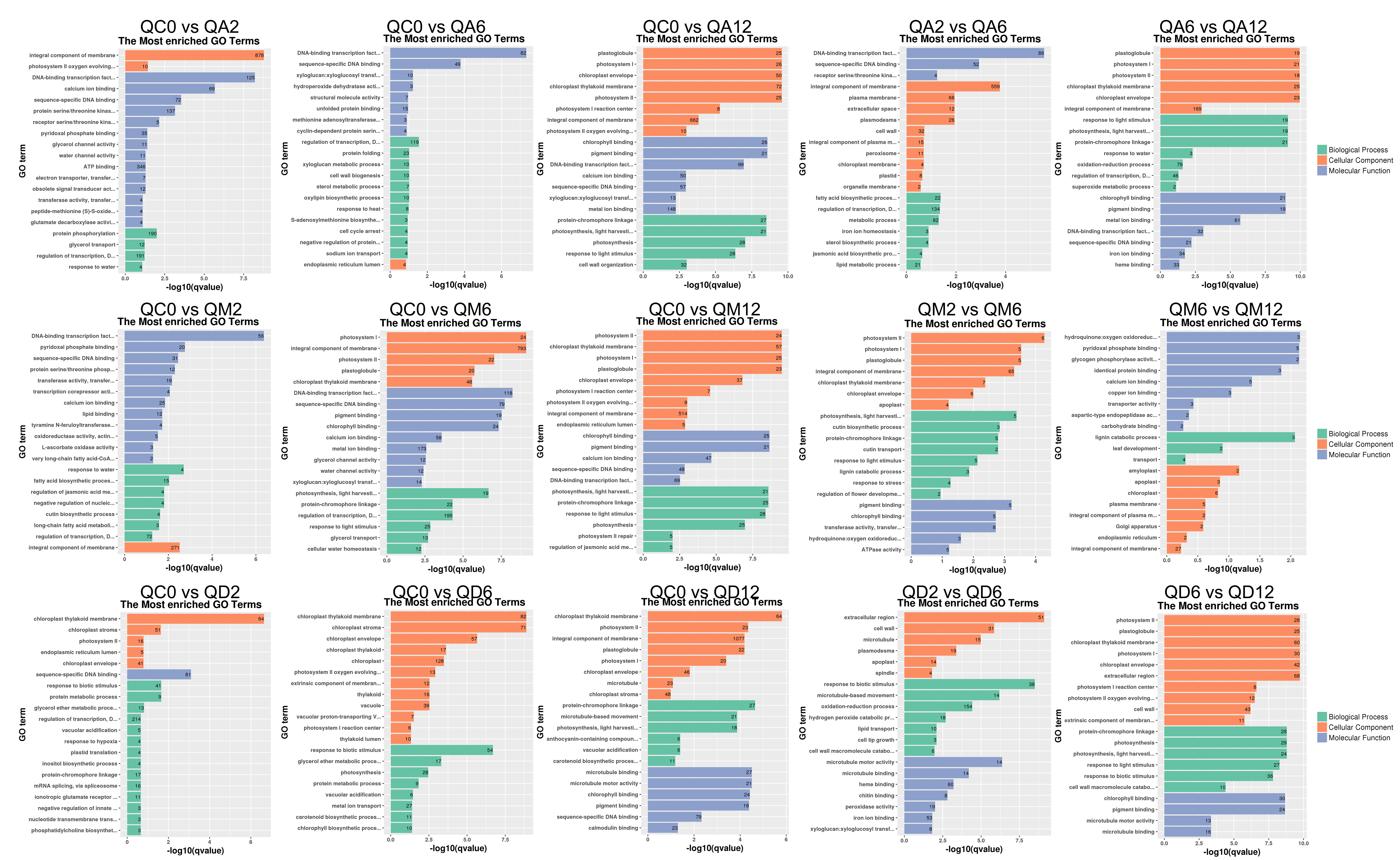

Supplement: Supplementary file 1 [file genes-13-02260-s001.zip › Supplementary Figure S7.tif]

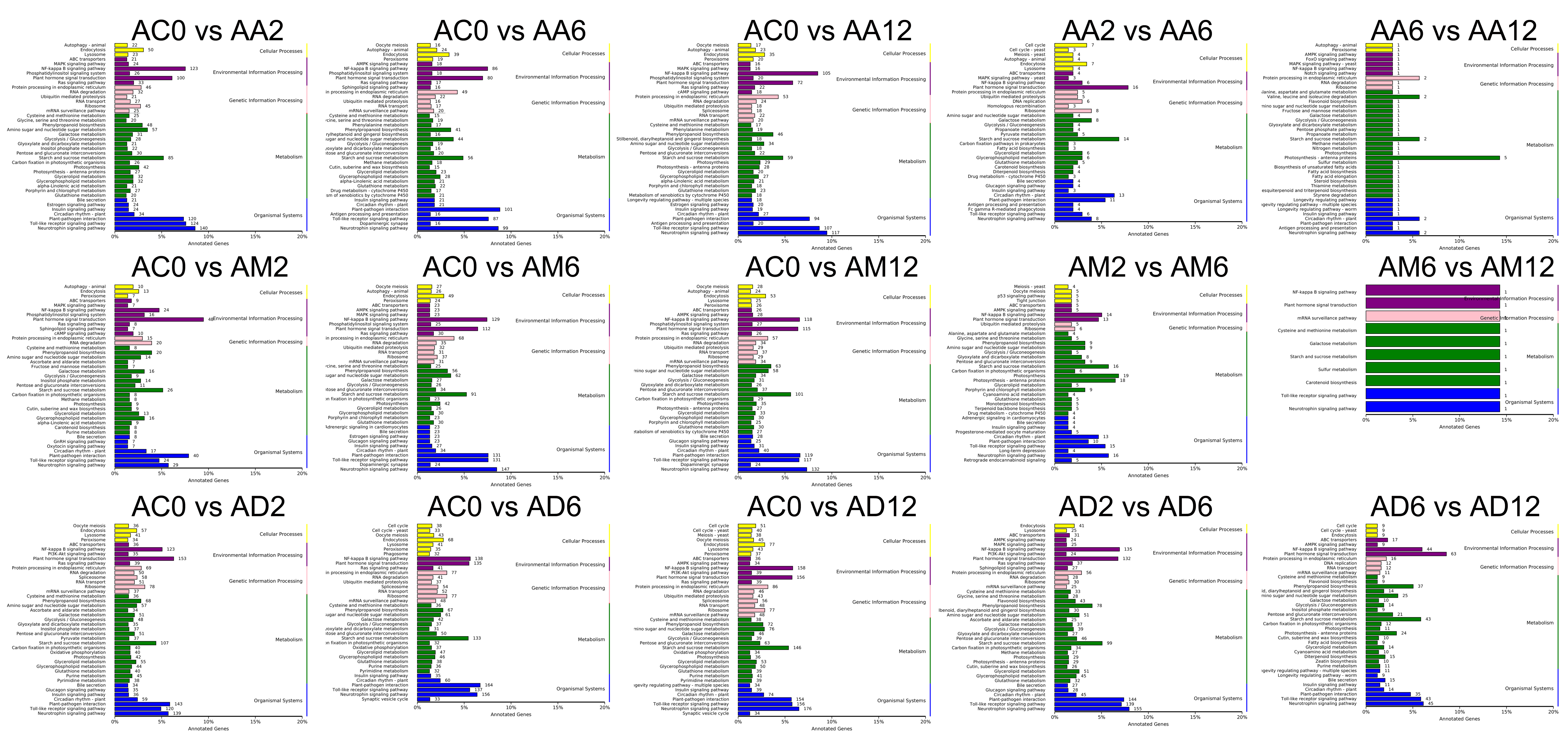

Supplement: Supplementary file 1 [file genes-13-02260-s001.zip › Supplementary Figure S8.tif]

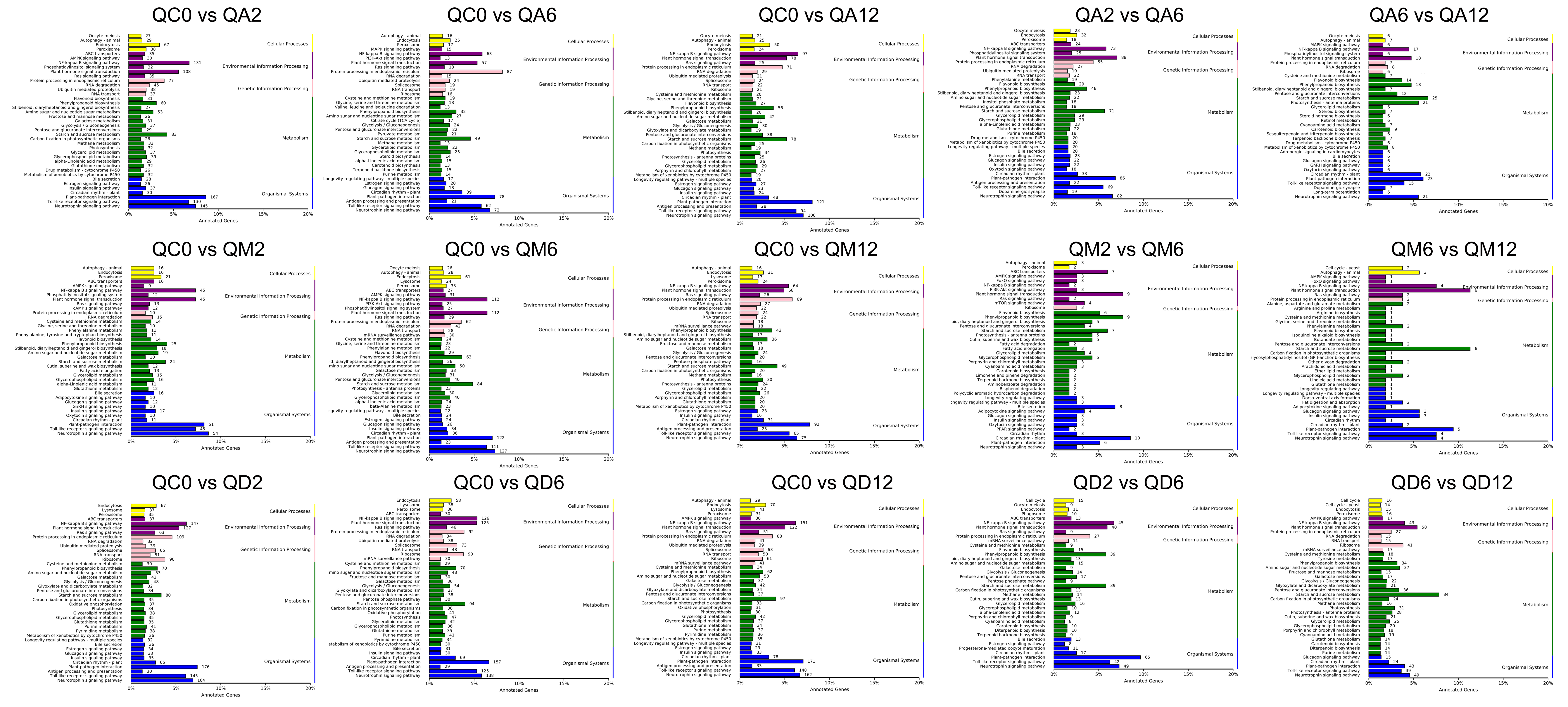

Supplement: Supplementary file 1 [file genes-13-02260-s001.zip › Supplementary Figure S9.tif]
